# A National Registry Study of Patient and Renal Survival in Adult Nephrotic Syndrome

**DOI:** 10.1016/j.ekir.2020.10.033

**Published:** 2020-11-04

**Authors:** Anna Kolb, Peter J. Gallacher, Jacqueline Campbell, Martin O’Neill, James R. Smith, Samira Bell, Bryan R. Conway, Wendy Metcalfe, Nicola Joss, Vishal Dey, Annette Alfonzo, Michael Kelly, Shahzad Shah, Emily McQuarrie, Colin Geddes, Jamie Traynor, Robert W. Hunter, Scottish Renal Biopsy Registry

**Affiliations:** 1 Department of Renal Medicine, Royal Infirmary of Edinburgh, Edinburgh Bioquarter, Edinburgh, Scotland, UK; 2 Centre for Cardiovascular Science, Queen’s Medical Research Institute, Edinburgh Bioquarter, University of Edinburgh, Edinburgh, Scotland, UK; 3 The Scottish Renal Registry, Scottish Health Audits, Public Health & Intelligence, Information Services, Glasgow, Scotland, UK; 4 Department of Renal Medicine, Aberdeen Royal Infirmary, Foresterhill Health Campus, Aberdeen, Scotland, UK; 5 Division of Population Health and Genomics, University of Dundee, Dundee, Scotland, UK; 6 Department of Renal Medicine, Raigmore Hospital, Inverness, Scotland, UK; 7 Department of Renal Medicine, University Hospital, Crosshouse, Kilmarnock, Scotland, UK; 8 Department of Renal Medicine, Victoria Hospital, Kirkcaldy, Scotland, UK; 9 Department of Renal Medicine, Dumfries & Galloway Royal Infirmary, Dumfries, Scotland, UK; 10 Department of Renal Medicine, University Hospital Monklands, Airdrie, Scotland, UK; 11 Glasgow Renal & Transplant Unit, Queen Elizabeth University Hospital, Glasgow, Scotland, UK

**Keywords:** cardiovascular disease, ESKD, glomerular disease, minimal-change nephropathy, mortality, nephrotic syndrome

## Abstract

**Introduction:**

We aimed to determine the mortality rate, cause of death, and rate of end-stage kidney disease (ESKD) in adults with nephrotic syndrome (NS).

**Methods:**

We conducted a national registry–based study, including all 522 adults who had a kidney biopsy for NS in Scotland in 2014–2017. We linked the Scottish Renal Registry to death certificate data. We performed survival and Cox proportional hazards analyses, accounting for competing risks of death and ESKD. We compared mortality rates with those in the age- and sex-matched general population.

**Results:**

A total of 372 patients had primary NS; 150 had secondary NS. Over a median follow-up of 866 days, 110 patients (21%) died. In patients with primary NS, observed versus population 3-year mortality was 2.1% (95% CI 0.0%–4.6%) versus 0.9% (0.8%–1.0%) in patients aged <60 years and 24.9% (18.4%–30.8%) versus 9.4% (8.3%–10.5%) in those aged ≥60 years. In secondary NS, this discrepancy was 17.1% (5.6%–27.2%) versus 1.1% (0.9%–1.2%) in <60-year-olds and 49.4% (36.6%–59.7%) versus 8.1% (6.6%–9.6%) in ≥60-year-olds. In primary NS, cardiovascular causes accounted for 28% of deaths, compared with 18% in the general population. Eighty patients (15%) progressed to ESKD. Incidence of ESKD by 3 years was 8.4% (95% CI 4.9%–11.7%) in primary and 35.1% (24.3%–44.5%) in secondary NS. Early remission of proteinuria and the absence of early acute kidney injury (AKI) were associated with lower rates of death and ESKD.

**Conclusions:**

Adults with NS have high rates of death and ESKD. Cardiovascular causes account for excess mortality in primary NS.

## Graphical abstract

**Figure:**
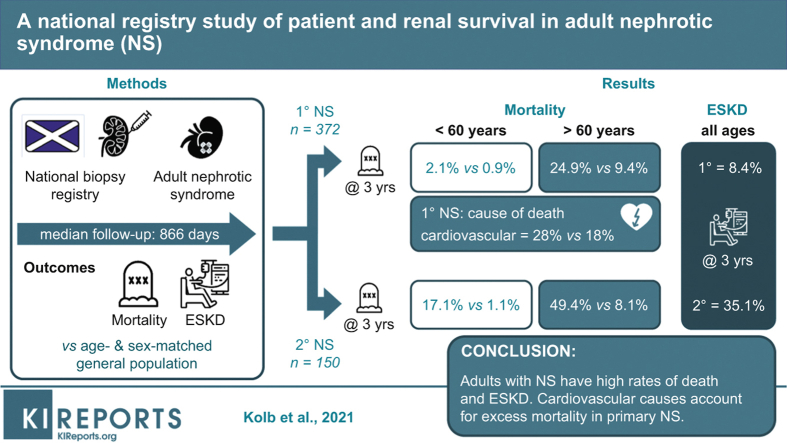

See Commentary on Page 246

The prognosis in adult NS is not well described in the literature, having been predominantly defined using small case series and in terms of short-term, disease-specific renal outcomes such as whether or not there is remission of proteinuria. NS is associated with progressive kidney failure, infection, thrombosis, and cancer.^1^^,^^2^ A few small observational studies in older adults have reported high death rates,^3^ but in general hard, patient-centered outcomes such as death and ESKD have not been well quantified.

To our knowledge, no nationwide studies have systematically evaluated patient-centered outcomes in adult NS. Indeed, a recent comprehensive review of minimal-change nephropathy did not mention risk of death or ESKD.^4^ We aimed to address this deficiency by performing a national, registry-based data linkage study of adults with biopsy-proven NS. We assessed mortality rates, cause of death, and rates of progression to ESKD.

## Methods

The methods are given in full in the Supplementary Methods.

### Design, Setting, and Participants

We conducted a retrospective, observational, registry-based cohort study including all adults who had a native kidney biopsy for NS in Scotland between January 1, 2014, and December 31, 2017. We retrieved records for biopsies in whom NS was deemed the indication for biopsy by local clinicians. Where patients had multiple biopsies during the study period, we included only data pertaining to the first biopsy. Follow-up was censored on December 31, 2018.

We prespecified subgroups of interest prior to retrieving any data. These were designed to draw comparisons by NS etiology (primary glomerular disease vs. secondary causes), diagnosis, age (18–59 vs. ≥60 years), and remission of proteinuria within the first 6 months. We published a prespecified analysis plan on April 2, 2019 (https://www.srr.scot.nhs.uk/Biopsy-Registry/_docs/OPRINEPH-protocol-paper.pdf). We have adhered to the principles of this plan but have deviated from the specifics in 2 respects. First, we were unable to obtain high-quality data on morbidity outcomes and drug prescription and have therefore not included these. Second, we had originally intended to present data for primary NS only, because of the heterogeneity in secondary NS. However, these data provide a useful context, and so we present data here for all NS.

### Variables, Data Sources, and Measurements

Our data sources were the National Records of Scotland for mortality data and the Scottish Renal Biopsy Registry for all other data.^5^ Individual patient data from these sources were linked through a unique national patient identifier (CHI number). The Scottish Renal Biopsy Registry records data for all adult kidney biopsies in Scotland.^6^

We retrieved data on demographics (age, sex, Scottish Index of Multiple Deprivation [SIMD] from SIMD16), disease (glomerular diagnosis), laboratory results (serum creatinine, serum albumin, urine protein-to-creatinine ratio, and urine albumin-to-creatinine ratio), and renal replacement therapy events (dialysis or kidney transplant). The SIMD is a validated measure of social deprivation derived from the patients’ address^7^; this is expressed in quintiles, with the lowest (ranked 1) representing areas with the greatest socioeconomic deprivation. Laboratory results were collected on the day of biopsy and after 1, 2, 3, 4, 6, 12, 18, and 24 months. Estimated glomerular filtration rate (eGFR) was calculated using the CKD-EPI equation.^8^

We retrieved the date and cause of death from the information entered on death certificates. We retrieved all of the causes of death entered in part I of the death certificate, which permits up to 4 causes to be listed. We classified cause of death using prespecified categories (Supplementary Table S1). Age- and sex-specific mortality rates in the general population were taken from publicly accessible national records.^9^

### Definition of Renal Outcomes

Renal survival was defined as the time to the first of any of the following events: starting dialysis, receiving a kidney transplant or an eGFR falling to <15 ml/min at any point during follow-up and then not returning to >15 ml/min at subsequent time points.

AKI during the first 4 months of follow-up was defined according to KDIGO serum creatinine criteria.^10^ We defined baseline creatinine as the lowest serum creatinine concentration within the first 3 months, to account for any AKI at the time of biopsy.

Remission of NS was classified according to KDIGO definitions.^11^ When defining remission within the first 6 months, we took the lowest urine protein-to-creatinine and albumin-to-creatinine ratios and the highest serum albumin result within the first 6 months.

### Statistical Methods

Data were analyzed in R (version 3.6.1).^12^ Our code is publicly accessible (https://github.com/robertwhunter/nephrotic_survival_SRR).

Standardized mortality was calculated as the ratio between the observed number of deaths and the number of deaths expected in the general population, given the age, sex, and follow-up duration for each patient.

Survival was determined using a survival (Kaplan-Meier) analysis. Survival was compared between prespecified subgroups using a Cox proportional hazards (CoxPH) model. We tested whether dependent variables satisfied the assumption of linearity. Age, eGFR, hemoglobin, and SIMD quintile were broadly linear and were therefore treated as continuous linear variables in the CoxPH model. Albumin had a nonlinear relationship with log hazard and was dichotomized in the final model. The urine protein-to-creatinine ratio did not predict mortality and was excluded from the final model. Renal survival was determined in a model that accounted for the competing risk of death. We use Fine and Gray’s method, having first excluded patients who had ESKD at baseline.^13^^,^^14^

## Results

### Participants

Across all 9 adult renal centers, there were 2811 native kidney biopsies in 2723 patients during the study period: an incidence of 130 biopsies per million population per year. A total of 556 patients were recorded in the registry as having had a biopsy for NS. We excluded 34 patients where case note review did not support a diagnosis of NS or where age was <18 years, leaving 522 patients in the final study cohort: an annual incidence of 24.2 per million population. Patients were followed up for a median 866 days (IQR 524–1264). The median number of glomeruli sampled in the formalin-fixed tissue was 13 (IQR 8–19). A minimum of 10 glomeruli were sampled in 69% of all biopsies.

Baseline demographic and laboratory data are presented in Table 1. Median age was 63.0 years (IQR 49.8–72.4); 46% were female. Overall, 372 patients had a primary glomerular cause of NS; 150 had a secondary cause. Patients with primary NS were drawn equally from across the socioeconomic spectrum (*P* = 0.53 for SIMD16 quintiles by χ^2^ test); patients with secondary NS were more likely to come from areas with higher socioeconomic deprivation (lower SIMD16 quintile), although this observation may have arisen through chance alone (*P* = 0.07).

**Table 1:** Baseline demographic and laboratory data

	All (*n* = 522)	Primary (*n* = 372)	Secondary (*n* = 150)
Demographic data			
Age, yr, median (IQR)	63.0 (49.8, 72.4)	63.5 (49.6, 72.5)	62.2 (50.5, 72.1)
Female	230 (46)	161 (45)	69 (48)
SIMD1	113 (22)	78 (21)	35 (24)
SIMD2	101 (20)	64 (17)	37 (25)
SIMD3	102 (20)	70 (19)	32 (22)
SIMD4	111 (22)	84 (23)	27 (18)
SIMD5	89 (17)	72 (20)	17 (11)
Laboratory data, median (IQR)			
Alb, g/l	22.0 (16.0, 26.0)	21.0 (15.0, 25.8)	23.0 (18.8, 27.2)
U.PCR, mg/mmol	810 (475, 1130)	812 (475, 1127)	804 (482, 1164)
U.ACR, mg/mmol	538 (327, 802)	572 (280, 829)	464 (336, 680)
Cr, μmol/l	98 (72, 160)	93 (69, 140)	121 (84, 196)
eGFR, ml/min	62 (35, 91)	70 (41, 93)	46 (28, 77)
Hb, g/dl	12.7 (10.8, 14.2)	13.0 (11.4, 14.4)	11.2 (9.7, 13.1)

Alb, serum albumin concentration; Cr, serum creatinine; eGFR, estimated glomerular filtration rate by CKD-EPI equation; Hb, hemoglobin concentration; IQR, interquartile range; U.ACR, urine albumin-to-creatinine ratio; U.PCR, urine protein-to-creatinine ratio.

SIMD1 to SIMD5 refer to Scottish Index of Multiple Deprivation quintiles, ranging from 1 (most socioeconomic deprivation) to 5 (least deprivation).

Values are *n* (%), unless otherwise noted.

There was little difference between the primary and secondary groups with respect to age, sex, serum albumin, or proteinuria. Patients with secondary NS had a higher serum creatinine (median 121 vs.93 μmol/l) and lower hemoglobin (median 11.2 vs. 13.0 g/l) at the time of biopsy.

Causative diagnoses, stratified by age, are presented in Supplementary Figure S1 and Supplementary Table S2. Membranous nephropathy, minimal-change nephropathy, and focal segmental glomerulosclerosis were the most common primary glomerular diagnoses; all 3 diagnoses were all more common in older adults. Diabetes, plasma cell dyscrasia, and systemic lupus erythematosus were the commonest causes of secondary NS.

### **Mortality** Rate

**Figure 1:**
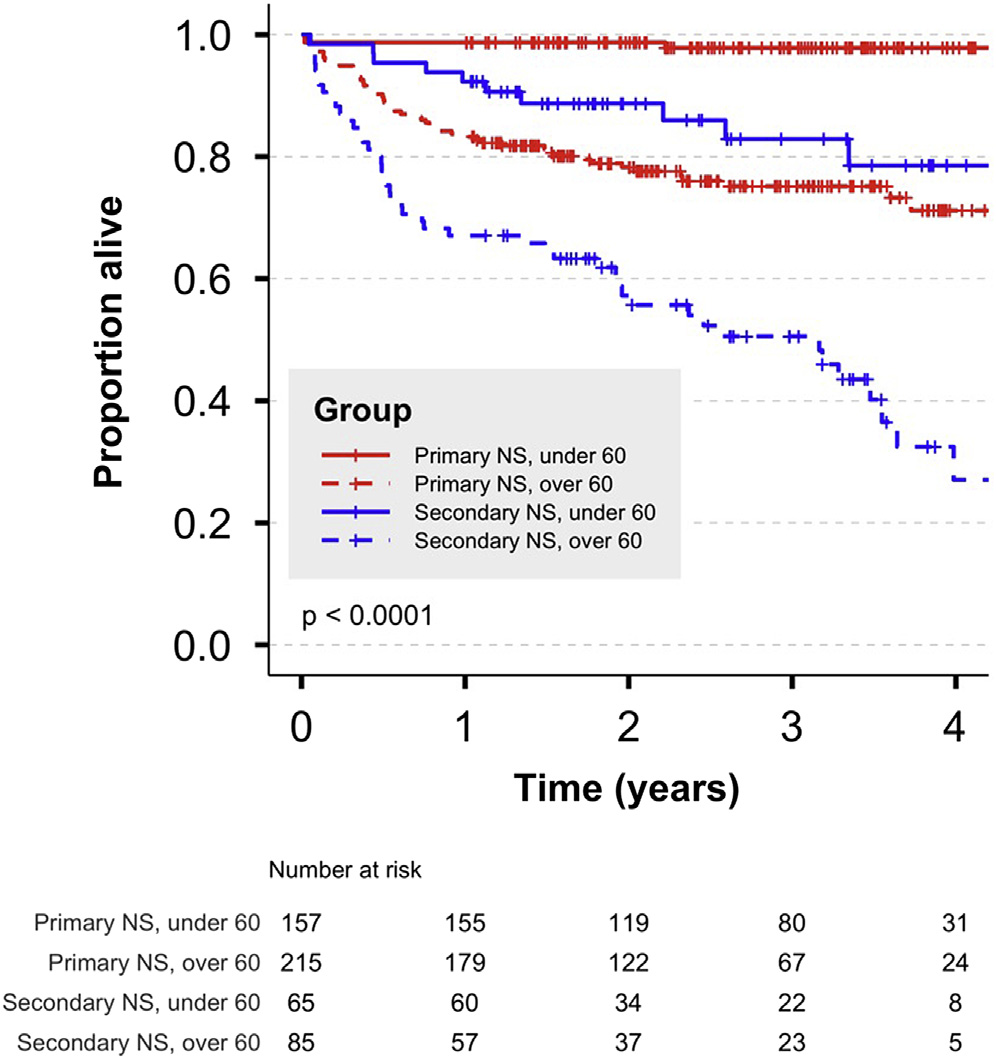
Patient survival curves, stratified by cause of nephrotic syndrome (NS) and age. Check-marks denote times at which patients were censored from the analysis (end of follow-up). *P* value is for comparison between groups by log-rank test.

One hundred ten patients (21%) died during follow-up. Death was more likely in patients with secondary NS and in those aged ≥60 years (Table 2; Figure 1) (*P* < 0.0001 by log-rank test). Observed mortality was higher than that predicted in the age- and sex-matched general population (Tables 3 and 4). The standardized mortality ratio, stratified by age and cause of NS, is shown in Table 3. To provide a clinically relevant assessment of absolute risk, we also calculated the 3-year mortality and compared this to predicted 3-year mortality in the age- and sex-matched general population (Table 4). In patients aged ≥60 years with primary NS, observed versus predicted 3-year mortality was 24.9% (95% CI 18.4%–30.8%) versus 9.4% (8.3%–10.5%). In secondary NS, this discrepancy was more pronounced, being 17.1% (5.6%–27.2%) versus 1.1% (0.9%–1.2%) in the <60-year-olds and 49.4% (36.6%–59.7%) versus 8.1% (6.6%–9.6%) in the ≥60-year-olds.

**Table 2:** Summary of patient-centered outcomes stratified by cause of nephrotic syndrome and age (<60 vs. ≥60 years)

	Whole cohort (*n* = 522)	Age <60 yr		Age ≥60 yr	
		Primary NS (*n* = 157)	Secondary NS (*n* = 65)	Primary NS (*n* = 215)	Secondary NS (*n* = 85)
Mortality and ESKD					
All death	110 (21)	3 (2)	10 (15)	51 (24)	46 (54)
All ESKD	80 (15)	13 (8)	19 (29)	22 (10)	26 (31)
ESKD then death	36 (7)	0 (0)	4 (6)	13 (6)	19 (22)
ESKD without death	44 (8)	13 (8)	15 (23)	9 (4)	7 (8)
Death without ESKD	74 (14)	3 (2)	6 (9)	38 (18)	27 (32)
Neither death nor ESKD	368 (70)	141 (90)	40 (62)	155 (72)	32 (38)
First ESKD event					
Dialysis	18 (23)	6 (46)	4 (21)	2 (10)	6 (23)
Transplant	0 (0)	0 (0)	0 (0)	0 (0)	0 (0)
eGFR <15	54 (68)	6 (46)	14 (74)	14 (67)	20 (77)
eGFR <15 at baseline	7 (9)	1 (8)	1 (5)	5 (24)	0 (0)
All ESKD events					
Dialysis only	42 (8)	9 (6)	11 (17)	10 (5)	12 (14)
Transplant only	1 (0)	0 (0)	0 (0)	1 (0)	0 (0)
Dialysis and transplant	11 (2)	4 (3)	5 (8)	0 (0)	2 (2)
eGFR <15 without RRT	26 (5)	0 (0)	3 (5)	11 (5)	12 (14)
None	442 (85)	144 (92)	46 (71)	193 (90)	59 (69)

ESKD, end-stage kidney disease; eGFR, estimated glomerular filtration rate by CKD-EPI equation; NS, nephrotic syndrome; RRT, renal replacement therapy.

Data are presented as *n* (%).

**Table 3:** Difference between observed and expected mortality: age- and sex-adjusted standardized mortality ratio, calculated over the duration of follow-up

Group	*n*	Deaths observed	Deaths expected	Standardized mortality ratio (95% CI)
Primary NS, age <60 yr	157	3	1.0	3.0 (1.0–9.4)
Primary NS, age ≥60 yr	215	51	9.7	5.2 (4.0–6.9)
Secondary NS, age <60 yr	65	10	0.4	24.2 (13.0–44.9)
Secondary NS, age ≥60 yr	85	46	3.0	15.4 (11.5–20.5)

CI, confidence interval; NS, nephrotic syndrome.

**Table 4:** Difference between observed and expected mortality: observed and expected 3-year mortality in the whole study cohort

Group	Observed 3**-**yr mortality (**mean** with 95% CI)	Predicted 3**-**yr mortality (mean with 95% CI)
Primary NS, age <60 yr	0.021 (0.000–0.046)	0.009 (0.008–0.010)
Primary NS, age ≥60 yr	0.249 (0.184–0.308)	0.094 (0.083–0.105)
Secondary NS, age <60 yr	0.171 (0.056–0.272)	0.011 (0.009–0.012)
Secondary NS, age ≥60 yr	0.494 (0.366–0.597)	0.081 (0.066–0.096)

CI, confidence interval; NS, nephrotic syndrome.

The observed 3-year mortality is presented (mean with 95% CI). The expected 3-year mortality in the age- and sex-matched general population was calculated for each cohort (mean with 95% CI).

**Figure 2:**
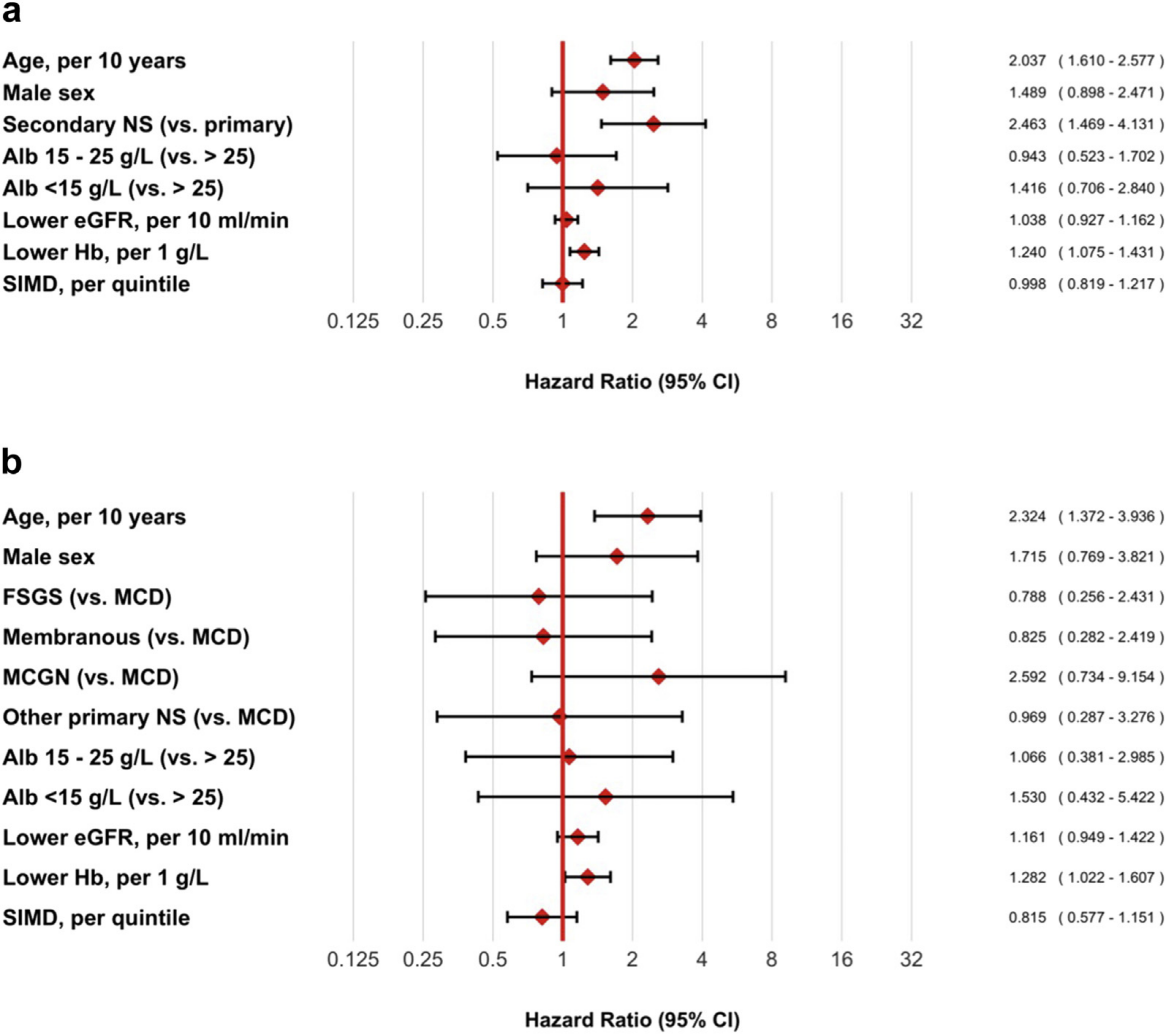
Cox proportional hazards models for risk of death. Multivariable models were constructed to test the association between predictor baseline variables and death for (a) the whole study cohort and (b) the subgroup with primary nephrotic syndrome aged ≥60 years. Alb, serum albumin concentration; eGFR, estimated glomerular filtration rate calculated by CKD-EPI equation; FSGS, focal segmental glomerulosclerosis; Hb, hemoglobin concentration; MCD, minimal-change nephropathy; MCGN, mesangiocapillary glomerulonephritis; NS, nephrotic syndrome; SIMD, Scottish Index of Multiple Deprivation.

In a CoxPH model, increasing age, secondary NS, and low baseline hemoglobin concentration were associated with mortality (Figure 2a). Risk of death in secondary NS may be largely determined by the underlying systemic disorder, and risk of death in younger patients with primary NS is very low. Therefore, we performed a prespecified analysis in patients aged ≥60 years with primary NS. In this model, increasing age (hazard ratio [HR] 2.32 per decade, 95% CI 1.37–3.94, *P* = 0.002) and lower hemoglobin concentration (HR 1.28 per g/l, 95% CI 1.02–1.61, *P* = 0.03) were associated with mortality; histologic diagnosis was not (Figure 2b; Supplementary Figure S2).

To explore which variables were most strongly associated with death or ESKD, we constructed sequentially adjusted models (Supplementary Table S3). The addition of SIMD quintile had little effect on model fit, as assessed using the Akaike information criterion, suggesting that it does not have a strong independent association with the risk of death or ESKD.

### Cause of Death

The causes of death are shown in Supplementary Table S4 and Supplementary Figure S3. We first considered only the single leading cause of death. In an attempt to minimize misclassification bias, we also performed a sensitivity analysis in which all causes of death for each patient were included. Patients with primary NS were most likely to die from cardiovascular causes, which were listed as the leading cause of death in 15 patients (28%) and as a contributory factor in 25 (46%). Patients with secondary NS were relatively less likely to die from cardiovascular disease and more likely to die from cancer. Venous thromboembolism was exceedingly uncommon as a cause of death, being listed as a contributory factor in a single patient with secondary NS.

### ESKD

**Figure 3:**
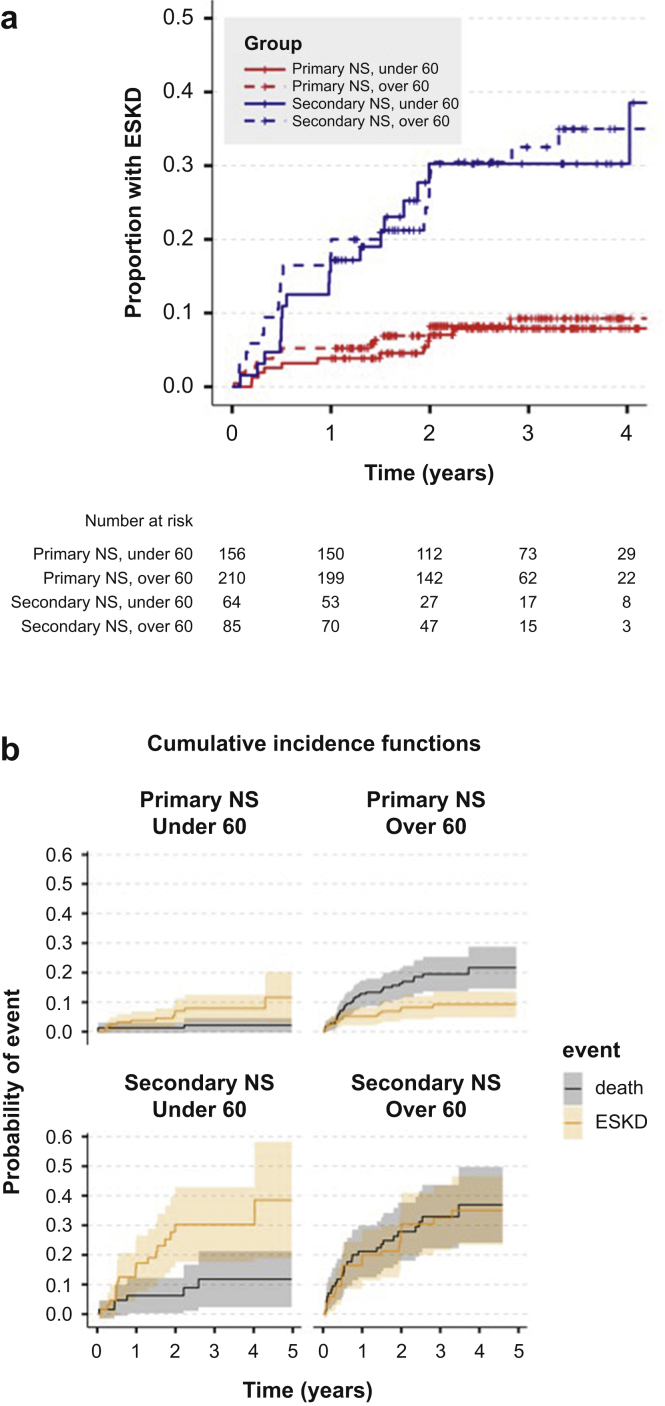
Proportion with end-stage kidney disease (ESKD), stratified by age and cause of nephrotic syndrome (NS). (a) Event curves were drawn using a Fine-Gray model, accounting for the competing risk of death. Patients were censored at the end of the follow-up period (check-marks). (b) Cumulative incidence of death or ESKD, stratified by age and cause of NS.

Eighty patients (15%) progressed to ESKD during follow-up. ESKD during follow-up was more common in patients with secondary than primary NS (Table 2; Figure 3a). There was a competing risk between progression to ESKD and death. This is evident in the cumulative incidence functions (Figure 3b): in patients aged <60 years, the risk of ESKD was higher than the risk of death. In patients aged ≥60 years, the risk of death was higher than that of ESKD in patients with primary NS and equivalent to the risk of ESKD in patients with secondary NS. In a model accounting for this competing risk, the rates of ESKD (with 95% CIs) at 3 years were 8.4% (4.9%–11.7%) in primary NS and 35.1% (24.3%–44.5%) in secondary NS.

**Figure 4:**
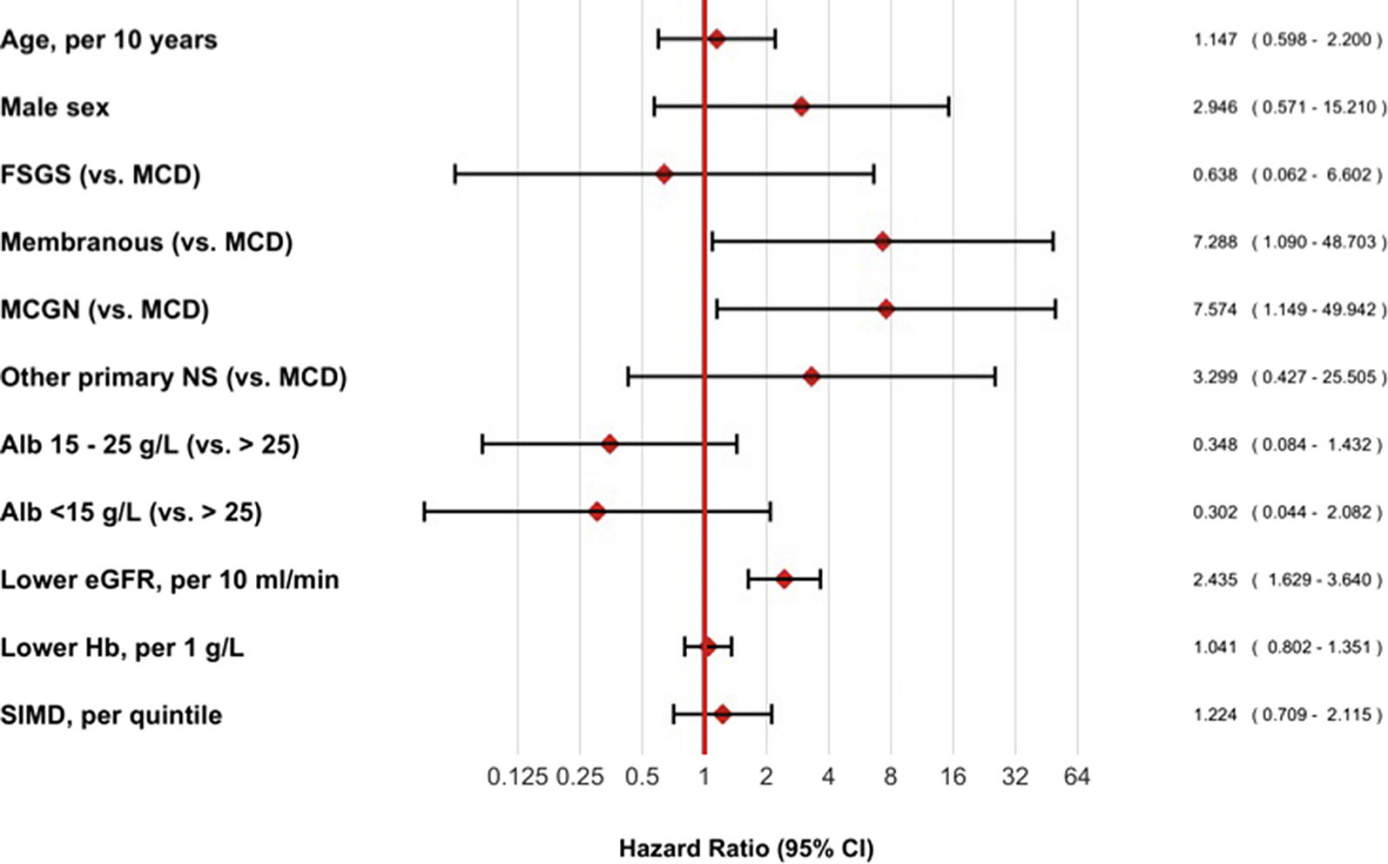
Cox proportional hazards model for risk of end-stage kidney disease (ESKD) in older patients with primary nephrotic syndrome (NS). A multivariable model was constructed to test the association between predictor baseline variables and the onset of ESKD for the cohort with primary NS aged ≥60 years. The model accounts for the competing risk of death. Alb, serum albumin concentration; eGFR, estimated glomerular filtration rate calculated by CKD-EPI equation; FSGS, focal segmental glomerulosclerosis; Hb, hemoglobin concentration; MCD, minimal-change nephropathy; MCGN, mesangiocapillary glomerulonephritis; SIMD, Scottish Index of Multiple Deprivation.

We constructed a multivariable model, accounting for the competing risk of death, in patients aged ≥60 years with primary NS (Figure 4). This subgroup analysis was prespecified for the reasons stated above. In this model, lower baseline eGFR was associated with risk of progression to ESKD (HR 2.4, 95% CI 1.6–3.6 per 10-ml increment in eGFR, *P* < 0.00001) as was a diagnosis of membranous nephropathy (HR 7.3, 95% CI 1.1–48.7 relative to minimal change, *P* = 0.04) or mesangiocapillary glomerulonephritis (HR 7.6, 95% CI 1.1–49.9 relative to minimal change, *P* = 0.04).

### Association of Remission With Death and ESKD

Laboratory data are presented in Supplementary Figure S4. Across the cohort, there was progressive reduction in proteinuria and an increase in serum albumin.

Overall, 238 (46%) patients achieved complete or partial remission within 6 months; 151 (29%) patients did not achieve even partial remission (Table 5). The remaining patients either died within the first 6 months or had missing follow-up proteinuria data.

**Table 5:** Remission data, stratified by cause of nephrotic syndrome and age

	Whole cohort (*n* = 522)	Age <60 yr		Age ≥60 yr	
		Primary NS (*n* = 157)	Secondary NS (*n* = 65)	Primary NS (*n* = 215)	Secondary NS (*n* = 85)
Remission by 6 mo					
Complete remission	76 (15)	33 (21)	3 (5)	39 (18)	1 (1)
Partial remission	162 (31)	65 (41)	16 (25)	62 (29)	19 (22)
Complete or partial	238 (46)	98 (62)	19 (29)	101 (47)	20 (24)
No remission	151 (29)	34 (22)	29 (45)	58 (27)	30 (35)
Data missing	65 (12)	18 (11)	9 (14)	26 (12)	12 (14)
Death before 6 mo	45 (9)	2 (1)	3 (5)	21 (10)	19 (22)

NS, nephrotic syndrome.

We determined the extent of any remission of nephrotic syndrome within 6 months of the biopsy date.

Data are presented as *n* (% of the total in that patient subgroup).

Within the cohort with primary NS, the frequency of achieving remission within 6 months was higher in those with a diagnosis other than membranous nephropathy, mesangiocapillary glomerulonephritis, or focal segmental glomerulosclerosis (Supplementary Table S5). We tested whether older age was associated with a reduced incidence of entering remission in primary NS by comparing patients aged <60 years with those aged ≥60 years. Overall, patients in the younger group achieved remission slightly earlier, but this difference was not statistically significant (Supplementary Figure S5A and B). Similarly, there were no statistically significant differences in time to remission in older versus younger patients for any of the individual primary glomerular diagnosis; the data for minimal-change nephropathy are presented in Supplementary Figure S5C and D.

In an analysis restricted to patients who survived 6 months after the index biopsy, entering early partial or complete remission was associated with a progressive reduction in the rates of death and progression to ESKD (Supplementary Figure S6).

### Association of AKI With Death and ESKD

Eighty-eight patients (17%) had an AKI at the time of kidney biopsy. In an analysis restricted to those patients who survived for more than 4 months after biopsy, 186 (39 %) developed an AKI during the first 4 months. AKI was more common in secondary NS and in patients aged ≥60 years (Supplementary Table S6). An early episode of AKI was associated with increased rates of death and progression to ESKD (Supplementary Figure S7).

## Discussion

### Summary and Perspective

We evaluated patient-centered outcomes in a national, registry-based study of adults who underwent kidney biopsy for NS.

We made 3 main observations. First, mortality in this patient group is significantly higher than in the age- and sex-matched general population. Three-year mortality was increased by 2–4-fold in primary and 6–16-fold in secondary NS. Cardiovascular deaths were relatively over-represented in primary NS. Second, there were high rates of progression to ESKD, particularly in secondary NS (in whom about one-third had developed ESKD within 3 years). In primary NS, baseline eGFR and glomerular diagnosis were associated with risk of ESKD. Third, failure to achieve early remission of NS and early AKI were both associated with higher mortality and an increased risk of ESKD.

We presented data for all adults who had a kidney biopsy for NS. The most valuable insights are probably drawn from older adults with primary NS. In secondary NS, it is hard to draw generalizable conclusions because clinical outcomes are driven by the causative systemic disease and because the patient population differs between centers depending on the prevalence of diabetes, lupus, infectious diseases, obesity etc. Death and ESKD are relatively uncommon in younger patients with primary NS. However, older patients (aged ≥60 years) with primary NS are a relatively homogenous group, in whom death and ESKD are common events. We therefore focused our analysis on this group and were able to draw conclusions that are likely to be generalizable.

### Death in Adult NS

We found that adults who underwent kidney biopsy for NS had a 3-year mortality rate that was significantly higher than in the age- and sex-matched general population. That this discrepancy was very large (up to 16-fold) in secondary NS is not surprising because the major causes of a secondary NS—diabetes, plasma cell dyscrasia, systemic lupus erythematosus—are all associated with reduced life-expectancy. The mortality risk associated with primary NS is noteworthy and has not been so clearly defined before.

Low baseline hemoglobin concentration was associated with an increased risk of death. It seems plausible that hemoglobin serves as a surrogate marker of poor health, rather than mediating a direct causal effect on mortality.

It is noteworthy that socioeconomic deprivation was not associated with an increased risk of death, in contrast to the strong association of socioeconomic deprivation and mortality in the general population.^15^ This suggests that the predominant causes of death in adults with NS override any socioeconomic drivers of disease and is in keeping with a model in which death is caused by aspects of the NS *per se*, its causative diseases, or its treatments.

Cardiovascular death was relatively over-represented in patients with primary NS, in whom it accounted for 28% of deaths as the leading cause. In the Scottish general population, cardiovascular disease (ischemic heart disease or cerebrovascular disease) accounted for 18% of deaths in 2018.^9^ Death from cancer was relatively less common—9% in primary NS versus 28% in the general population—presumably predominantly because of the competing risk of cardiovascular death.

With respect to cause of death, there were 2 noteworthy “relevant negatives.” The proportion of deaths caused by cancer in the cohort with secondary NS (29%) was similar to that in the general population. Although a Danish cohort study reported an association between NS and a diagnosis of cancer,^2^ our data suggest that cancer is not hugely over-represented as a *cause of death* in adults with NS. Similarly, despite the association of NS with venous thromboembolism,^16^ we found that this was a very infrequent cause of death. We were not able to evaluate the frequency of nonfatal venous thromboembolism, nor to factor in the effect of antithrombotic prophylaxis.

### Renal Outcomes in Adult NS

Progression to ESKD was a relatively rare event in primary NS (∼8% by 3 years) but occurred frequently (∼35% by 3 years) in secondary NS. These data, although not surprising given the natural history of the diseases that cause NS, could help to inform treatment planning decisions.

By 6 months, patients were alive and in complete or partial remission of NS in 53% (primary NS) or 26% (secondary NS). Only 50% of adults with minimal-change nephropathy entered complete remission within 6 months. This underscores the relatively treatment-resistant nature of adult minimal-change nephropathy and is worse than the remission rates reported elsewhere.^17^^,^^18^ One potential explanation for this discrepancy is that our population have characteristics that are associated with an unfavorable disease outcome. For example, our minimal-change nephropathy group (median age 63 years) was older than many published cohorts.^17^, ^18^, ^19^, ^20^ Another potential explanation is that using our registry-based approach, we included patients who were not treated with immunosuppressive therapies: a group frequently excluded from observational studies. It is also possible that we have misclassified patients as having minimal-change nephropathy when they actually had focal segmental glomerulosclerosis, but unlikely that this will be the case for many given the generally adequate glomerular sampling (>10 glomeruli in 69% of all biopsies). In historic case series, remission of minimal-change nephropathy is delayed in adults aged ≥60 years.^3^ We made a similar observation, although this was reasonably likely to have occurred through chance alone in our data set (*P* > 0.05).

These poor outcomes, relative to childhood NS, may reflect differences in physiological reserve or the underlying glomerular disease. Minimal-change nephropathy in children typically remits within days of starting glucocorticoid treatment, whereas in adults it often takes months to respond or fails to remit at all.^21^

Unsurprisingly, entering early remission was associated with lower mortality and reduced progression to end-stage kidney disease. This is merely an observation; only well-conducted RCTs can tell us whether therapies that achieve early remission also improve these hard clinical outcomes.

Within the first 4 months, 39% of our study cohort developed AKI: a result broadly consistent with the literature.^22^^,^^23^ Early AKI was associated with progression to ESKD and death. Even mild (stage 1) AKI was associated with mortality, suggesting that this may reflect confounding differences in patient characteristics that predispose both to AKI and to death. On the other hand, the risk of death was higher for severe (stage 3) AKI, suggesting that there may also be a causal relationship between AKI and death.

### Strengths

Our study has all the strengths inherent in a large, registry-based, multicenter study. We used data linkage to ensure that we captured date and cause of death completely and accurately.

In the largest existing study of mortality in NS, cases were identified retrospectively using death certificate data to find patients who had died from NS.^24^ However, in our study only 20 death certificates (18%) included NS as a contributory cause of death and only 71 (65%) included any renal cause. Therefore, any study using death certificate data to identify NS will miss at least 35% of cases. Our prospective, registry-based identification of cases is better placed to provide a complete picture of the risk and cause of death in adult NS. Our cause of death data may be subject to misclassification bias. However, we guarded against that by using the most accurate source of data (i.e., death certificates) and by including a sensitivity analysis in which the top 4 causes of death were evaluated and not just the leading cause.

### Limitations

We acknowledge all the general limitations inherent in an observational study in addition to the following specific limitations.

Although we collected national data over several years, there were small numbers in some of our subgroups of interest. In our CoxPH models (Figures 2 and 4), this limits the precision of our HR estimates and increases the likelihood of a type II error (i.e., we may have been underpowered to detect real associations between patient characteristics and mortality or ESKD). On the other hand, in testing multiple covariates, we may have observed associations that occurred through chance alone (type I error). For example, it will be interesting to see whether our observed association between primary glomerular diagnosis and risk of ESKD is replicated in other, larger populations.

Furthermore, in our CoxPH models, we selected covariates *a priori* as being potentially clinically relevant with respect to the outcomes of interest (death and ESKD). However, we were limited to variables for which we had good-quality data; it is likely that other unmeasured characteristics such as smoking history would exhibit stronger associations with these outcomes.

We compared our mortality data to the age- and sex-matched general population, thus not accounting for many other potential confounding variables. The Scottish population is predominantly white Caucasian, and our findings may not be generalizable to other ethnic groups.

We only studied biopsy-proven NS. Primary NS can, by definition, only be diagnosed by kidney biopsy. However, many cases of suspected secondary NS are not investigated with kidney biopsy, particularly in the context of diabetes. Therefore, our findings in biopsied secondary NS are almost certainly not generalizable to all patients with secondary NS.

We lack additional data that would help to interpret our main findings. We were not able to capture robust data sets pertaining to morbidity outcomes and treatment (including immunosuppression and thromboprophylaxis).

We chose a definition of AKI that relied solely on the serum creatinine rising (above standard thresholds) in the first 4 months. We did not require any subsequent recovery of renal function, as incorporating this into the definition could have introduced a survivorship bias, moving any association of AKI with death toward the null. Therefore, our definition of AKI may have included some patients who had rapid, irreversible progression of CKD. If so, one might expect any bias to result in our overestimating the associations of AKI with death and ESKD.

### Implications

Our findings should be used to help patients and their clinicians to make treatment decisions. Planning for ESKD management (renal replacement therapy or conservative care) should be actively pursued, particularly in patients with secondary NS. Advance care planning for end-of-life care may be appropriate, particularly in older patients and even in primary glomerular diseases like minimal-change nephropathy. We have demonstrated that minimal-change nephropathy is not the benign condition that it is often considered to be.

Our findings also have implications for our understanding of the pathophysiology of NS and for future research. In the cohort with primary NS, the uniformly poor survival across disease subgroups suggests that premature death is caused by NS *per se* or by the treatment for the underlying glomerular disease. The excess deaths were due to cardiovascular causes. The potential drivers of this association are myriad: deleterious sequelae of chronic volume overload, hyperlipidemia or steroid therapy causing accelerated atherosclerosis, electrolyte disturbance causing dysrhythmia, etc. Future mechanistic and epidemiologic studies should attempt to determine the dominant causative pathways. Furthermore, the excess of deaths from cardiovascular causes provides a rationale for interventional studies testing cardiovascular risk reduction strategies in this patient group.

### Conclusions

Adults with NS, investigated by kidney biopsy, have increased rates of death and ESKD. Failure to achieve early remission and early AKI are associated with death and ESKD. In primary NS, cardiovascular deaths are over-represented compared to the general population.

## Disclosure

All the authors declared no competing interests.

## References

[1] Hull R.P., Goldsmith D.J.A. (2008). Nephrotic syndrome in adults. BMJ.

[2] Christiansen C.F., Onega T., Sværke C. (2014). Risk and prognosis of cancer in patients with nephrotic syndrome. Am J Med.

[3] Nolasco F., Stewart Cameron J., Heywood E.F. (1986). Adult-onset minimal change nephrotic syndrome: a long-term follow-up. Kidney Int.

[4] Vivarelli M., Massella L., Ruggiero B., Emma F. (2017). Minimal change disease. Clin J Am Soc Nephrol.

[5] The Scottish Renal Registry Scottish renal biopsy registry. https://www.srr.scot.nhs.uk/Biopsy-Registry/Main.html.

[6] McQuarrie E.P., Mackinnon B., Young B. (2009). Centre variation in incidence, indication and diagnosis of adult native renal biopsy in Scotland. Nephrol Dial Transplant.

[7] Scottish Index of Multiple Deprivation 2020—gov.scot. https://www.gov.scot/collections/scottish-index-of-multiple-deprivation-2020/.

[8] Levey A.S., Stevens L.A., Schmid C.H. (2009). A new equation to estimate glomerular filtration rate. Ann Intern Med.

[9] National Records of Scotland 2013. https://www.nrscotland.gov.uk/statistics-and-data/statistics/statistics-by-theme/vital-events/general-publications/vital-events-reference-tables/2018/section-6-death-causes.

[10] Thomas M.E., Blaine C., Dawnay A. (2015). The definition of acute kidney injury and its use in practice. Kidney Int.

[11] Kidney Disease: Improving Global Outcomes (KDIGO) Glomerulonephritis Work Group (2012). KDIGO clinical practice guideline for glomerulonephritis. Kidney Int Suppl.

[12] R Core Team (2019). R: A Language and Environment for Statistical Computing. https://www.R-project.org/.

[13] Therneau T., Crowson C., Atkinson E. Multi-state models and competing risks. https://cran.r-project.org/web/packages/survival/vignettes/compete.pdf.

[14] Fine J.P., Gray R.J. (1999). A proportional hazards model for the subdistribution of a competing risk. J Am Stat Assoc.

[15] Fenton L., Wyper G.M., McCartney G., Minton J. (2019). Socioeconomic inequality in recent adverse all-cause mortality trends in Scotland. J Epidemiol Commun Health.

[16] Kerlin B.A., Ayoob R., Smoyer W.E. (2012). Epidemiology and pathophysiology of nephrotic syndrome-associated thromboembolic disease. Clin J Am Soc Nephrol.

[17] Waldman M., Crew R.J., Valeri A. (2007). Adult minimal-change disease: clinical characteristics, treatment, and outcomes. Clin J Am Soc Nephrol.

[18] Maas R.J., Deegens J.K., Beukhof J.R. (2017). The clinical course of minimal change nephrotic syndrome with onset in adulthood or late adolescence: a case series. Am J Kidney Dis.

[19] Gipson D.S., Troost J.P., Lafayette R.A. (2016). Complete remission in the nephrotic syndrome study network. Clin J Am Soc Nephrol.

[20] Fernandez-Juarez G., Villacorta J., Ruiz-Roso G. (2016). Therapeutic variability in adult minimal change disease and focal segmental glomerulosclerosis. Clin Kidney J.

[21] Hogan J., Radhakrishnan J. (2013). The treatment of minimal change disease in adults. J Am Soc Nephrol.

[22] Fujigaki Y., Tamura Y., Nagura M. (2017). Unique proximal tubular cell injury and the development of acute kidney injury in adult patients with minimal change nephrotic syndrome. BMC Nephrol.

[23] Meyrier A., Niaudet P. (2018). Acute kidney injury complicating nephrotic syndrome of minimal change disease. Kidney Int.

[24] Wakasugi M., Kazama J.J., Narita I. (2018). Premature mortality due to nephrotic syndrome and the trend in nephrotic syndrome mortality in Japan, 1995-2014. Clin Exp Nephrol.

