# Supplementary material for: A National Registry Study of Patient and Renal Survival in Adult Nephrotic Syndrome
Source: Kidney Int Rep. 2020 Nov 4;6(2):449–59. doi: 10.1016/j.ekir.2020.10.033 (PMC7879209; doi:10.1016/j.ekir.2020.10.033)
Supplement: Supplementary File (PDF) [file mmc1.pdf]

A national registry study of patient and renal survival in adult nephrotic syndrome

Kolb et al. KI Reports

Supplemental material

Table of contents

1) supplemental methods
2) supplemental table & figure legends
3) supplemental tables (S1 – S6)
4) supplemental figures (S1 – S7)
5) supplemental references
6) STROBE statement

Supplemental methods

Design, setting & participants

We conducted a retrospective, observational, registry-based cohort study including all adults who had a native kidney biopsy for NS in Scotland between 1st January 2014 and 31st December 2017. We retrieved records for biopsies in whom NS was deemed the indication for biopsy by local clinicians. Two physicians independently reviewed each patient's case notes, clinical and laboratory data. Where this information did not support a diagnosis of NS, patients were excluded from the study. Where patients had multiple biopsies during the study period, we included only data pertaining to the first biopsy. Follow-up was censored on 31st December 2018.

We pre-specified subgroups of interest prior to retrieving any data. These were designed to draw comparisons by NS aetiology (primary glomerular disease *vs.* secondary causes), diagnosis, age (18–59 *vs.* over 60 years) and remission of proteinuria within the first 6 months. We published a pre-specified analysis plan on 2nd April 2019 (https://www.srr.scot.nhs.uk/Biopsy-Registry/_docs/OPRINEPH-protocol-paper.pdf). We have adhered to the principles of this plan but have deviated from the specifics in two respects. First, we were unable to obtain high-quality data on morbidity outcomes and drug prescription and have therefore not included these. Second, we had originally intended to present data for primary NS only, because of the heterogeneity in secondary NS. However, these data provide a useful context and so we present data here for all NS.

Data were handled securely in accordance with Caldicott principles and with the approval of NHS National Services Scotland.

Variables, data sources and measurements

Our data sources were the National Records of Scotland for mortality data and the Scottish Renal Biopsy Registry for all other data (1). Individual patient data from these sources were linked through a unique national patient identifier (CHI number). The Scottish Renal Biopsy Registry records data for all adult kidney biopsies in Scotland (2).

We retrieved data on demographics (age, sex, Scottish Index of Multiple Deprivation from SIMD16), disease (glomerular diagnosis), laboratory results (serum creatinine [SCr], serum albumin, urine protein:creatinine ratio [uPCR] and urine albumin:creatinine ratio [uACR]) and renal replacement therapy events (dialysis or kidney transplant). The Scottish Index of Multiple Deprivation (SIMD) is a validated measure of social deprivation derived from the patients' address (3); this is expressed in quintiles with the lowest (1) representing areas with the greatest socioeconomic deprivation.

Laboratory results were collected on the day of biopsy and after 1, 2, 3, 4, 6, 12, 18 and 24 months. For each time-point, we took the result closest to the relevant date, accepting any result within 30 days either side. Estimated glomerular filtration rate (eGFR) was calculated using the CKD-EPI equation (4). Laboratory data were censored after the start of dialysis therapy or kidney transplant.

From the National Records of Scotland, we retrieved the date and cause of death from the information entered on death certificates. We retrieved all of the causes of death entered in part I of the death certificate, which permits up to four causes to be listed. We classified cause of death using pre-specified categories (supplemental Table S1). We compared

observed three-year mortality with predicted three-year mortality in the matched general population, calculating the latter from age and sex-specific mortality rates published by National Records Scotland (5).

Definition of renal outcomes

Renal survival was defined as the time to the first of any of the following events: starting dialysis, receiving a kidney transplant or an eGFR falling to <15 ml/min at any point during follow-up and then not returning to >15 ml/min at subsequent time-points.

Acute kidney injury (AKI) during the first four months of follow-up was defined according to KDIGO serum creatinine criteria (6). We defined baseline creatinine as the lowest serum creatinine concentration within the first three months, to account for any AKI at the time of biopsy.

Remission of NS was classified according to KDIGO definitions (7). Complete remission was defined as uPCR <30 mg/mmol (265 mg/g) and a serum albumin >35 g/L; partial remission as uPCR 30–350 mg/mmol (265–3097 mg/g). Where we had data for uACR instead, these thresholds were 20 and 220 mg/mmol (177 and 1947 mg/g) respectively. When defining remission within the first 6 months, we took the lowest uPCR/uACR and the highest serum albumin result within the first 6 months.

Statistical methods

Data were analysed in R (version 3.6.1) (8) using the *Tidyverse* package (version 1.3.0) (9). Our code is publicly accessible (https://github.com/robertwhunter/nephrotic_survival_SRR).

Standardised mortality was calculated as the ratio between the observed number of deaths and the number of expected deaths in the general population, given the age, sex and follow-up duration for each patient.

Survival was determined using a survival (Kaplan-Meier) analysis using the *Survival* (version 3.1-12) and *Survminer* (version 0.4.6) packages (10,11). Survival was compared between pre-specified subgroups using a Cox proportional hazards (Cox PH) model. We tested whether dependent variables satisfied the assumption of linearity. Age, eGFR, haemoglobin and SIMD quintile were broadly linear and were therefore treated as continuous linear variables in the Cox PH model. Albumin had a non-linear relationship with log hazard and was dichotomised in the final model. uPCR did not predict mortality and was excluded from the final model. In the final models, Schoenfeld residuals supported an assumption of proportional hazards.

Renal survival was determined in a model that accounted for the competing risk of death. We use Fine & Gray's method, having first excluded patients who had ESKD at baseline (12,13). We confirmed the accuracy of our competing risks model by constructing an alternative model using the *cmprsk* package (version 2.2-10) (14).

When evaluating outcomes stratified by remission of NS within the first 6 months, we excluded all patients who died or reached ESKD within the first 6 months. When evaluating outcomes stratified by AKI within the first 4 months, we excluded all patients who died or reached ESKD within the first 4 months.

Supplemental Table & Figure legends

Table S1: Cause of death coding. Causes of death were classified into pre-specified categories. The individual causes of death assigned to each category heading are listed here.

Table S2: Age distribution of glomerular and systemic diagnoses causing nephrotic syndrome. Abbreviations: FSGS, focal segmental glomerulosclerosis; MCGN, mesangiocapillary glomerulonephritis; IgAN, IgA nephropathy. These same data are presented in graphical form in Figure S1.

Table S3: Sequentially adjusted CoxPH models. These tables demonstrate the effect of adding covariates to the CoxPH models. The Akaike information criterion (AIC) provides a measure of how well each model represents that data and allows comparison between alternative models; a lower AIC is usually preferable. **A)** Mortality in the whole cohort. **B)** Mortality in patients with primary nephrotic syndrome aged over 60. **C)** ESKD, accounting for the competing risk of death, in patients with primary nephrotic syndrome aged over 60.

Table S4: Cause of death. Causes of death. In the upper table, the leading single cause of death for each patient is represented. In the bottom table, any contributing cause of death listed on the death certificate is included for each patient. Abbreviations: NS, nephrotic syndrome; VTE, venous thromboembolism; ESKD, end-stage kidney disease.

Table S5: Remission rate in primary nephrotic syndrome, stratified by age and primary renal disease. Remission within the first six months was assessed within the cohort of patients with primary nephrotic syndrome. Abbreviations: FSGS, focal segmental glomerulosclerosis; MCGN, mesangiocapillary glomerulonephritis; NS, nephrotic syndrome

Table S6: Acute kidney injury. Frequency of AKI, graded using the KDIGO serum creatinine criteria, during the first four months of follow-up. Abbreviations: AKI, acute kidney injury; 1ry NS, primary nephrotic syndrome; 2ry NS, secondary nephrotic syndrome.

Figure S1: Age distribution of glomerular and systemic diagnoses causing nephrotic syndrome. Abbreviations: FSGS, focal segmental glomerulosclerosis; MCGN, mesangiocapillary glomerulonephritis; IgAN, IgA nephropathy. These same data are presented in tabular form in Table S2.

Figure S2: Patient survival in the subgroup aged over 60 with primary nephrotic syndrome, stratified by glomerular diagnosis. Tick-marks denote times at which a patients were censored from the analysis (end of follow-up). p-value is for comparison between groups by logrank test. Abbreviations: FSGS, focal segmental glomerulosclerosis; MCGN, mesangiocapillary glomerulonephritis; NS, nephrotic syndrome.

Figure S3: Leading cause of death, stratified by age and cause of nephrotic syndrome. The leading cause of death was determined for every patient and classified into eight categories. Abbreviations: NS, nephrotic syndrome; ESKD, end-stage kidney disease; VTE, venous thromboembolism.

Figure S4: Laboratory data. A) Serum albumin; **B)** eGFR; **C) & D)** uPCR. In A, B and C, data are stratified by cause of nephrotic syndrome; in D by whether patients entered remission within the first six months. Abbreviations: Alb, serum albumin concentration; eGFR, estimated glomerular filtration rate by CKD-EPI equation; uPCR, urine protein:creatinine ratio; NS, nephrotic syndrome.

Figure S5: Time to remission of nephrotic syndrome, stratified by age. Time to **A)** complete or **B)** partial remission in patients with primary nephrotic syndrome. Time to **C)** complete or **D)** partial remission in patients with primary nephrotic syndrome caused by minimal change nephropathy. Patients were censored at death or the onset of end-stage kidney disease (tick-marks). p-value is for comparison between groups by logrank test.

Figure S6: Association of early remission with patient survival and progression to ESKD. Patients were classified according to whether or not their nephrotic syndrome entered remission within the first

six months of follow-up. Association of entering early remission with patient survival in **A)** primary nephrotic syndrome and **B)** secondary nephrotic syndrome. Association of early remission with ESKD in a model accounting for the competing risk of death in **C)** primary nephrotic syndrome and **D)** secondary nephrotic syndrome. p-value is for comparison between groups by logrank test. Abbreviations: uPCR, urine protein:creatinine ratio; ESKD, end-stage kidney disease.

Figure S7: Association of early AKI with patient survival and progression to ESKD. Patients were classified according to the worst grade of acute kidney injury during the first four months of follow-up. Association of early AKI with patient survival in **A)** primary nephrotic syndrome and **B)** secondary nephrotic syndrome. Association of early AKI with ESKD in a model accounting for the competing risk of death in **C)** primary nephrotic syndrome and **D)** secondary nephrotic syndrome. In secondary nephrotic syndrome, there were small numbers of patients with AKI so results were lumped into two groups (AKI of any stage *vs* no AKI). p-value is for comparison between groups by logrank test. Abbreviations: AKI, acute kidney injury; ESKD, end-stage kidney disease.

Supplemental Table S1

Cancer
diffuse non-hodgkin’s lymphoma
lymphoid leukaemia
malignant neoplasm of bladder
malignant neoplasm of breast
malignant neoplasm of bronchus and lung
malignant neoplasm of ovary
malignant neoplasm of prostate
malignant neoplasm of rectosigmoid junction
malignant neoplasm of rectum
malignant neoplasm of unspecified site
malignant neoplasms of lymphoid/haematopoietic/related tissue
malignant neoplasms of mesothelial and soft tissue
malignant neoplasms of thyroid and other endocrine glands
multiple myeloma and malignant plasma cell neoplasms
other and unspecified types of non-hodgkin’s lymphoma
other neo of uncert/unkn behav of lymphoid/haematopoietic/related tissue
secondary and unspecified malignant neoplasm of lymph nodes
secondary malignant neoplasm of other sites
secondary malignant neoplasm of respiratory and digestive organs

Cardiovascular
acute myocardial infarction
atrial fibrillation and flutter
cardiac arrest
cardiomyopathy
cerebral infarction
chronic ischaemic heart disease
complications and ill-defined descriptions of heart disease
diseases of arteries, arterioles and capillaries

heart failure
hypertensive diseases
hypertensive heart disease
ischaemic heart diseases
nonrheumatic aortic valve disorders
other acute ischaemic heart diseases
other aneurysm
other cardiac arrhythmias
other disorders of arteries and arterioles
other peripheral vascular diseases
pulmonary heart disease and diseases of pulmonary circulation
sequelae of cerebrovascular disease
stroke, not specified as haemorrhage or infarction

**ESKD**
renal failure

**Bleeding**
haemorrhage, not elsewhere classified
intracerebral haemorrhage

**Infection**
bacterial infection of unspecified site
cellulitis
influenza and pneumonia
other local infections of skin and subcutaneous tissue
other septicaemia
pneumonia, organism unspecified
streptococcal septicaemia
tuberculosis of other organs

**Nephrotic syndrome**

nephrotic syndrome

Other
abnormalities of gait and mobility
acc pois/exposure to narcotics & psychodysleptics not elsewhere classified
acc pois/exposure to other & unspec chemicals & noxious substances
acute pancreatitis
amyloidosis
asphyxiation
diabetes mellitus
diseases of myoneural junction and muscle
diseases of spleen
disorders of gallbladder, biliary tract and pancreas
disorders of mineral metabolism
disorders of muscles
diverticular disease of intestine
enlarged lymph nodes
extrapyramidal and movement disorders
fibrosis and cirrhosis of liver
fracture of femur
gangrene, not elsewhere classified
hormones, & their synthetic substitutes & antagonists, nec
inflammatory disorders of male genital organs, not elsewhere classified
intentional self-harm by hanging, strangulation and suffocation
med proc as cause of abn reac of pat/later comp w/o ment at time of proced
mental and behavioural disorders due to use of tobacco
mental/behav disorders due to mult drug & other psychoactive substuse
non-insulin-dependent diabetes mellitus
oedema, not elsewhere classified
other arthrosis
other chronic obstructive pulmonary disease
other diseases of biliary tract

other diseases of digestive system

other diseases of intestine

other diseases of intestines

other diseases of pancreas

other diseases of pleura

other disorders involving the immune mechanism, not elsewhere classified

other disorders of brain

other disorders of urinary system

other general symptoms and signs

other hypothyroidism

other ill-defined and unspecified causes of mortality

other inflammatory liver diseases

other interstitial pulmonary diseases

other metabolic disorders

other necrotizing vasculopathies

other rheumatoid arthritis

other soft tissue disorders, not elsewhere classified

other specified diabetes mellitus

paralytic ileus and intestinal obstruction without hernia

pneumoconiosis due to asbestos and other mineral fibres

pneumonitis due to solids and liquids

pois/expos to antiepilep/sed-hypntic/antipark/psychotrop drugs, undeterm

poisoning by antiepileptic, sedative-hypnotic and antiparkinsonism drugs

poisoning by hormones and their synthetic substitutes and antagonists, nec

poisoning by narcotics and psychodysleptics [hallucinogens]

poisoning by primarily systemic and haematological agents, nec

polycythaemia vera

primarily systemic agents

pulmonary oedema

recurrent and persistent haematuria

systemic atrophies primarily affecting the central nervous system

thalassaemia

toxic effect of other inorganic substances
unspecified acute lower respiratory infection
unspecified diabetes mellitus
vascular dementia

**Renal disease (other)**
chronic nephritic syndrome
chronic renal failure
hypertensive renal disease
other disorders of fluid, electrolyte and acid-base balance
other disorders of kidney and ureter, not elsewhere classified
tubulo-interstitial nephritis, not specified as acute or chronic
unspecified contracted kidney
unspecified nephritic syndrome
unspecified renal failure

**Thrombosis**
arterial embolism and thrombosis

Supplemental Table S2

Age (yrs)	Other primary NS	Membranous	MCGN	FSGS	Minimal change
< 30	9	4	3	5	10
30 - 40	5	7	0	6	9
40 - 50	5	11	1	8	13
50 - 60	7	22	3	12	17
60 - 70	8	41	4	19	27
70 - 80	8	35	3	14	19
> 80	3	9	3	8	14

Age (yrs)	Adaptive FSGS	Lupus	Diabetes	Other	2ry IgAN	Plasma cell dyscrasia	2ry MCGN	2ry membranous
< 30	1	5	1	0	0	0	0	0
30 - 40	0	3	5	2	0	0	0	0
40 - 50	0	6	7	2	2	2	0	0
50 - 60	0	6	7	4	1	9	1	1
60 - 70	3	5	11	4	1	14	0	2
70 - 80	5	0	7	9	1	12	2	1
> 80	1	0	0	2	0	5	0	0

Supplemental Table S3

A) Mortality in whole cohort | Final model is presented in Fig2A

	**Model 1**			**Model 2**			**Model 3**			**Model 4**			**Model 5**			**Model 6**		
Predictors	*Estimates*	*CI*	*p*	*Estimates*	*CI*	*p*	*Estimates*	*CI*	*p*	*Estimates*	*CI*	*p*	*Estimates*	*CI*	*p*	*Estimates*	*CI*	*p*
Age (per year increase)	1.08	1.06 – 1.10	**<0.001**	1.08	1.06 – 1.10	**<0.001**	1.07	1.05 – 1.09	**<0.001**	1.07	1.05 – 1.10	**<0.001**	1.07	1.05 – 1.10	**<0.001**	1.07	1.05 – 1.10	**<0.001**
Male sex	1.31	0.87 – 1.98	0.199	1.26	0.84 – 1.91	0.267	1.24	0.82 – 1.88	0.313	1.32	0.81 – 2.15	0.262	1.49	0.90 – 2.47	0.120	1.49	0.90 – 2.47	0.123
Secondary NS (vs. primary)				3.55	2.36 – 5.32	**<0.001**	3.08	2.02 – 4.70	**<0.001**	2.79	1.70 – 4.59	**<0.001**	2.47	1.49 – 4.07	**<0.001**	2.46	1.47 – 4.13	**0.001**
eGFR (per 1ml/min increase)							0.99	0.98 – 0.99	**0.001**	0.99	0.98 – 1.00	**0.007**	1.00	0.99 – 1.01	0.518	1.00	0.99 – 1.01	0.521
Albumin 15-25g/L (vs. >25g/L)										1.08	0.60 – 1.93	0.803	0.94	0.52 – 1.70	0.846	0.94	0.52 – 1.70	0.846
Albumin <15 g/L (vs. >25g/L)										1.55	0.78 – 3.10	0.212	1.41	0.71 – 2.82	0.329	1.42	0.71 – 2.84	0.327
Haemoglobin (per 1g/L increase)													0.81	0.70 – 0.93	**0.003**	0.81	0.70 – 0.93	**0.003**
SIMD (per quintile)																1.00	0.82 – 1.22	0.988
Observations	505			505			486			346			318			317		
AIC	1047.984			1014.009			964.589			685.552			651.919			653.842		

B) Mortality in primary NS aged over 60 | Final model is presented in Fig2B

	**Model 1**			**Model 2**			**Model 3**			**Model 4**			**Model 5**			**Model 6**		
Predictors	*Estimates*	*CI*	*p*	*Estimates*	*CI*	*p*	*Estimates*	*CI*	*p*	*Estimates*	*CI*	*p*	*Estimates*	*CI*	*p*	*Estimates*	*CI*	*p*
Age (per year increase)	1.11	1.06 – 1.16	**<0.001**	1.09	1.04 – 1.14	**<0.001**	1.09	1.04 – 1.14	**0.001**	1.08	1.03 – 1.14	**0.002**	1.09	1.04 – 1.15	**0.001**	1.09	1.03 – 1.15	**0.002**
Male sex	1.47	0.78 – 2.77	0.236	1.71	0.89 – 3.28	0.109	1.74	0.82 – 3.68	0.151	1.75	0.81 – 3.79	0.154	1.61	0.75 – 3.49	0.225	1.71	0.77 – 3.82	0.187
eGFR (per 1ml/min increase)				0.97	0.96 – 0.99	**<0.001**	0.97	0.96 – 0.99	**0.002**	0.98	0.96 – 1.00	0.084	0.98	0.96 – 1.00	0.068	0.99	0.97 – 1.01	0.147
Albumin 15-25g/L (vs. >25g/L)							1.12	0.41 – 3.03	0.828	0.97	0.35 – 2.68	0.951	0.96	0.35 – 2.65	0.937	1.07	0.38 – 2.98	0.903
Albumin <15 g/L (vs. >25g/L)							0.90	0.28 – 2.94	0.867	1.05	0.32 – 3.46	0.937	1.24	0.37 – 4.18	0.730	1.53	0.43 – 5.42	0.510
Haemoglobin (per 1g/L increase)										0.78	0.64 – 0.96	**0.021**	0.78	0.63 – 0.97	**0.024**	0.78	0.62 – 0.98	**0.032**
SIMD (per quintile)													0.78	0.56 – 1.08	0.130	0.82	0.58 – 1.15	0.245
FSGS (vs. MCD)																0.79	0.26 – 2.43	0.679
Membranous (vs. MCD)																0.83	0.28 – 2.42	0.727
MCGN (vs. MCD)																2.59	0.73 – 9.15	0.139
Other primary NS (vs. MCD)																0.97	0.29 – 3.28	0.959
Observations	206			196			141			127			127			127		
AIC	405.766			368.003			276.826			260.350			259.966			265.198		

C) ESKD in primary NS aged over 60 | Final model is presented in Fig4

	**Model 1**			**Model 2**			**Model 3**			**Model 4**			**Model 5**			**Model 6**		
Predictors	*Estimates*	*CI*	*p*	*Estimates*	*CI*	*p*	*Estimates*	*CI*	*p*	*Estimates*	*CI*	*p*	*Estimates*	*CI*	*p*	*Estimates*	*CI*	*p*
Age (per year increase)	1.04	0.98 – 1.11	0.207	0.98	0.91 – 1.05	0.635	1.00	0.94 – 1.08	0.883	1.00	0.93 – 1.08	0.902	1.00	0.92 – 1.09	0.960	1.01	0.93 – 1.10	0.679
Male sex	1.10	0.42 – 2.90	0.842	1.32	0.48 – 3.65	0.587	2.38	0.69 – 8.18	0.225	2.40	0.70 – 8.22	0.212	2.43	0.70 – 8.45	0.195	2.95	0.72 – 11.97	0.197
eGFR (per 1ml/min increase)				0.94	0.91 – 0.97	**<0.001**	0.93	0.90 – 0.97	**<0.001**	0.94	0.90 – 0.98	**0.001**	0.94	0.90 – 0.98	**<0.001**	0.91	0.86 – 0.97	**<0.001**
Albumin 15-25g/L (vs. >25g/L)							0.29	0.07 – 1.11	0.062	0.25	0.06 – 0.97	**0.038**	0.25	0.06 – 0.98	**0.042**	0.35	0.07 – 1.71	0.143
Albumin <15 g/L (vs. >25g/L)							0.21	0.04 – 1.15	0.161	0.18	0.03 – 1.09	0.147	0.18	0.03 – 1.08	0.146	0.30	0.05 – 1.94	0.224
Haemoglobin (per 1g/L increase)										0.96	0.71 – 1.30	0.742	0.96	0.71 – 1.30	0.734	0.96	0.68 – 1.36	0.762
SIMD (per quintile)													1.03	0.66 – 1.61	0.890	1.22	0.71 – 2.10	0.468
FSGS (vs. MCD)																0.64	0.06 – 6.58	0.706
Membranous (vs. MCD)																7.29	1.06 – 50.10	**0.040**
MCGN (vs. MCD)																7.57	0.75 – 76.57	**0.035**
Other primary NS (vs. MCD)																3.30	0.52 – 20.83	0.253
Observations	317			306			225			211			211			211		
AIC	177.539			142.913			118.686			117.996			119.980			120.144		

Supplemental Table S4

Leading cause of death	Primary NS, under 60 yrs	Primary NS, over 60 yrs	Secondary NS, under 60 yrs	Secondary NS, over 60 yrs	TOTAL
cardiovascular	1	14	2	6	23
renal disease (other)	0	6	0	2	8
cancer	0	5	3	13	21
infection	0	6	0	5	11
nephrotic syndrome	0	5	0	0	5
ESKD	0	0	0	0	0
bleeding	0	0	0	1	1
VTE	0	0	0	1	1
other	2	15	5	18	40
TOTAL	3	51	10	46	110

All causes of death	Primary NS, under 60 yrs	Primary NS, over 60 yrs	Secondary NS, under 60 yrs	Secondary NS, over 60 yrs	TOTAL
cardiovascular	2	23	6	21	52
renal disease (other)	1	25	1	21	48
cancer	0	11	5	20	36
infection	0	15	2	9	26
nephrotic syndrome	1	14	1	4	20
ESKD	1	1	0	1	3
bleeding	0	1	0	2	3
VTE	0	0	0	1	1
other	2	32	8	38	80
TOTAL	7	122	23	117	269

Supplemental Table S5

	Diagnosis: FSGS	Diagnosis: MCGN	Diagnosis: Membranous	Diagnosis: Minimal change	Diagnosis: Other primary NS
**Remission by 6 months (ALL)**					
n =	72	17	129	109	45
Complete remission (%)	11 (15)	0 (0)	1 (1)	54 (50)	6 (13)
Partial remission (%)	24 (33)	5 (29)	51 (40)	23 (21)	24 (53)
Complete or partial (%)	35 (49)	5 (29)	52 (40)	77 (71)	30 (67)
No remission (%)	14 (19)	4 (24)	57 (44)	12 (11)	5 (11)
Data missing (%)	17 (24)	2 (12)	13 (10)	9 (8)	3 (7)
Death before 6 months (%)	4 (6)	4 (24)	2 (2)	8 (7)	5 (11)
ESKD before 6 months (%)	2 (3)	2 (12)	5 (4)	3 (3)	2 (4)
**Remission by 6 months (AGE UNDER 60)**					
n =	31	7	44	49	26
Complete remission (%)	3 (10)	0 (0)	0 (0)	28 (57)	2 (8)
Partial remission (%)	12 (39)	3 (43)	19 (43)	12 (24)	19 (73)
Complete or partial (%)	15 (48)	3 (43)	19 (43)	40 (82)	21 (81)
No remission (%)	8 (26)	1 (14)	19 (43)	5 (10)	1 (4)
Data missing (%)	6 (19)	1 (14)	4 (9)	4 (8)	3 (12)

Death before 6 months (%)	1 (3)	1 (14)	0 (0)	0 (0)	0 (0)
ESKD before 6 months (%)	1 (3)	1 (14)	2 (5)	0 (0)	1 (4)
**Remission by 6 months (AGE OVER 60)**					
n =	41	10	85	60	19
Complete remission (%)	8 (20)	0 (0)	1 (1)	26 (43)	4 (21)
Partial remission (%)	12 (29)	2 (20)	32 (38)	11 (18)	5 (26)
Complete or partial (%)	20 (49)	2 (20)	33 (39)	37 (62)	9 (47)
No remission (%)	6 (15)	3 (30)	38 (45)	7 (12)	4 (21)
Data missing (%)	11 (27)	1 (10)	9 (11)	5 (8)	0 (0)
Death before 6 months (%)	3 (7)	3 (30)	2 (2)	8 (13)	5 (26)
ESKD before 6 months (%)	1 (2)	1 (10)	3 (4)	3 (5)	1 (5)

Supplemental Table S6

	Whole cohort	Under 60, 1ry NS	Under 60, 2ry NS	Over 60, 1ry NS	Over 60, 2ry NS
**AKI at baseline**					
n =	522	157	65	215	85
AKI all (%)	88 (17)	18 (11)	8 (12)	52 (24)	10 (12)
AKI1 (%)	59 (11)	13 (8)	7 (11)	30 (14)	9 (11)
AKI2 (%)	16 (3)	4 (3)	1 (2)	11 (5)	0 (0)
AKI3 (%)	13 (3)	1 (1)	0 (0)	11 (5)	1 (1)
**Worst AKI during early follow-up (first 4 months)**					
n =	472	150	60	195	67
AKI all (%)	186 (39)	40 (27)	26 (43)	83 (43)	37 (55)
AKI1 (%)	138 (29)	31 (21)	21 (35)	55 (28)	31 (46)
AKI2 (%)	34 (7)	7 (5)	4 (7)	18 (9)	5 (7)
AKI3 (%)	14 (3)	2 (1)	1 (2)	10 (5)	1 (1)

Figure S1

A

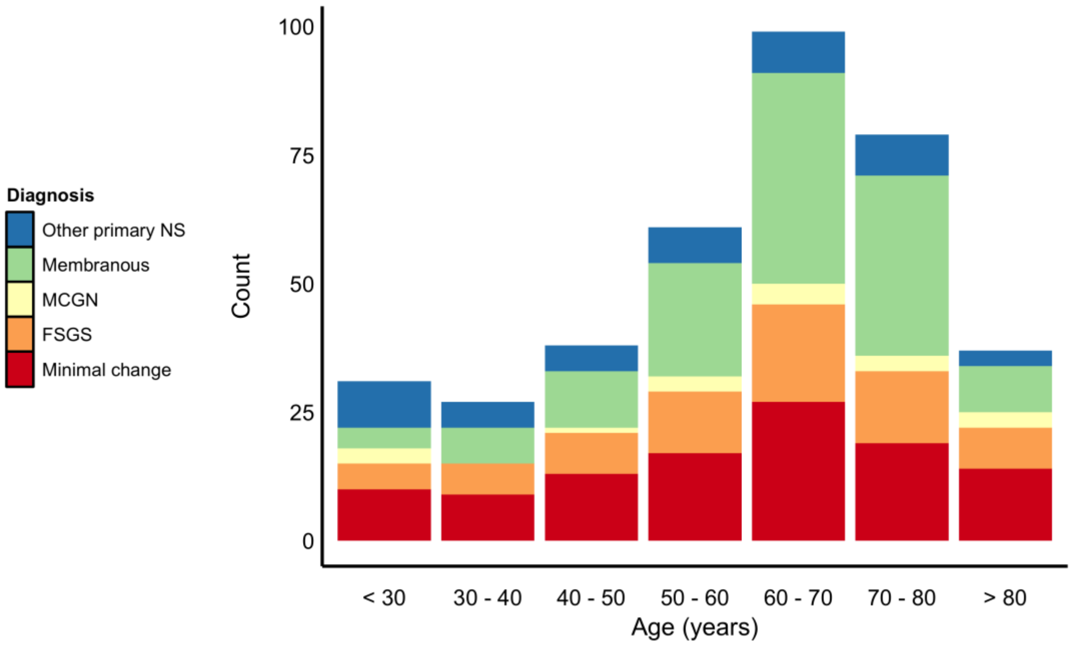
Diagnosis
Other primary NS
Membranous
MCGN
FSGS
Minimal change
Count
100
75
50
25
0
< 30
30 - 40
40 - 50
50 - 60
60 - 70
70 - 80
> 80
Age (years)

B

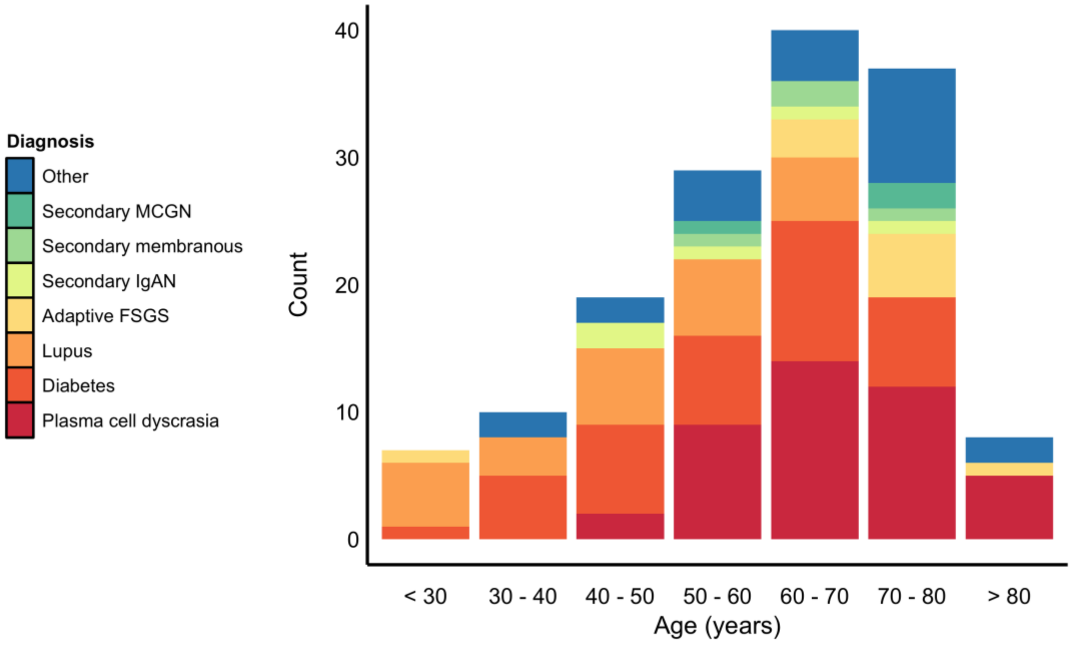
Diagnosis
Other
Secondary MCGN
Secondary membranous
Secondary IgAN
Adaptive FSGS
Lupus
Diabetes
Plasma cell dyscrasia
Count
40
30
20
10
0
< 30
30 - 40
40 - 50
50 - 60
60 - 70
70 - 80
> 80
Age (years)

Figure S2

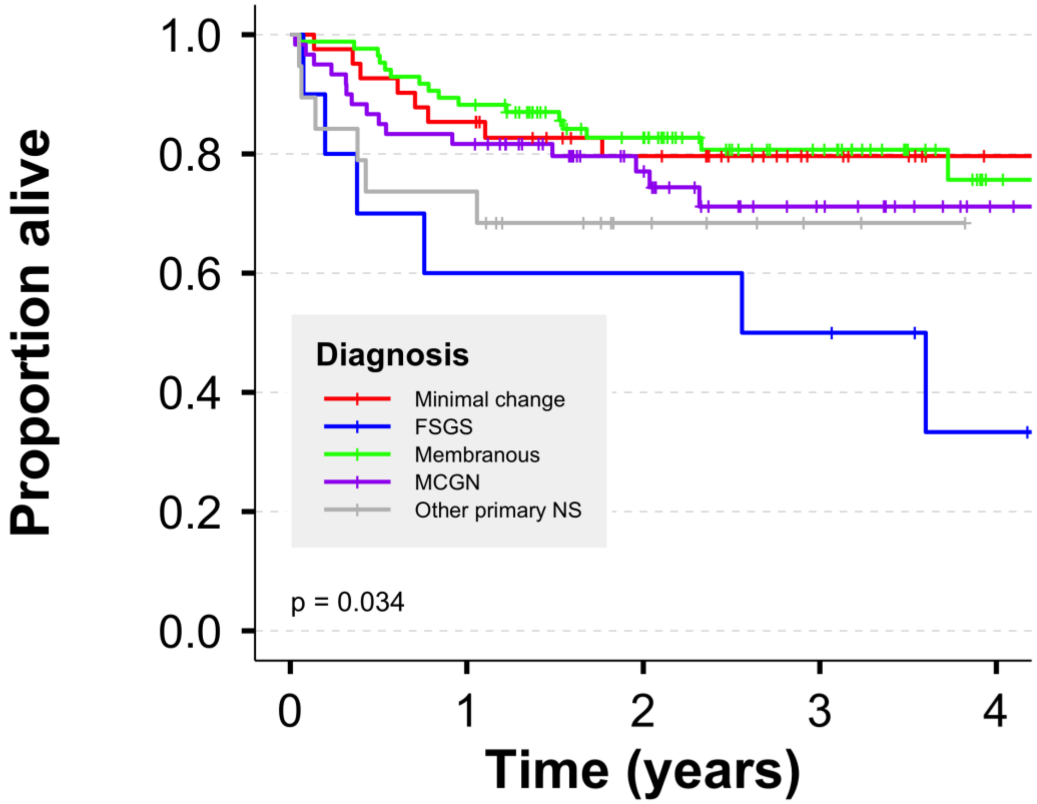

Number at risk

Minimal change	41	35	26	14	7
FSGS	10	6	6	5	2
Membranous	85	75	54	31	10
MCGN	60	49	30	15	5
Other primary NS	19	14	6	2	0

Figure S3

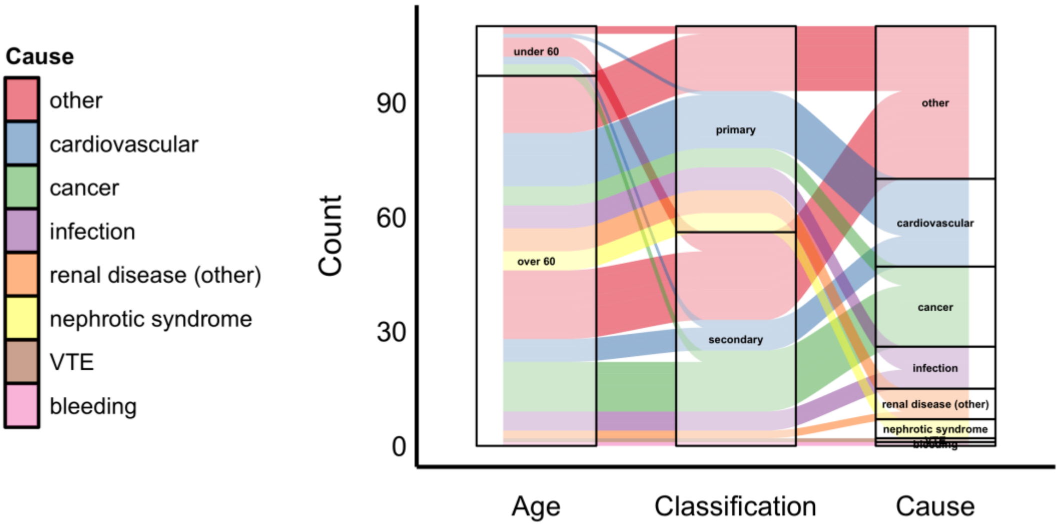
Cause
other
cardiovascular
cancer
infection
renal disease (other)
nephrotic syndrome
VTE
bleeding
Count
90
60
30
0
under 60
over 60
primary
secondary
other
cardiovascular
cancer
infection
renal disease (other)
nephrotic syndrome
Age
Classification
Cause

Figure S4

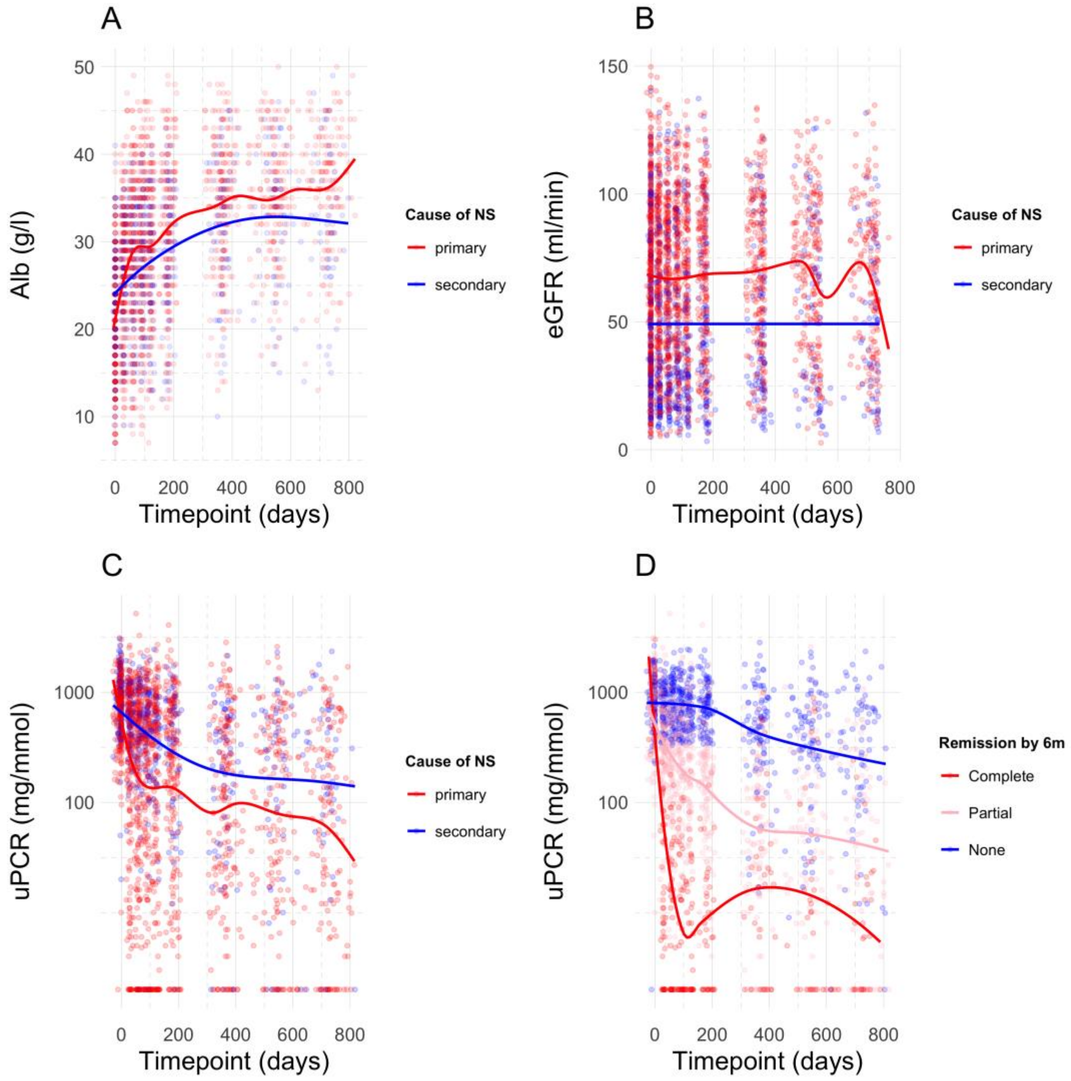
A
Alb (g/l)
50
40
30
20
10
0
200
400
600
800
Timepoint (days)
Cause of NS
primary
secondary
B
eGFR (ml/min)
150
100
50
0
0
200
400
600
800
Timepoint (days)
Cause of NS
primary
secondary
C
uPCR (mg/mmol)
1000
100
0
200
400
600
800
Timepoint (days)
Cause of NS
primary
secondary
D
uPCR (mg/mmol)
1000
100
0
200
400
600
800
Timepoint (days)
Remission by 6m
Complete
Partial
None

Figure S5

A

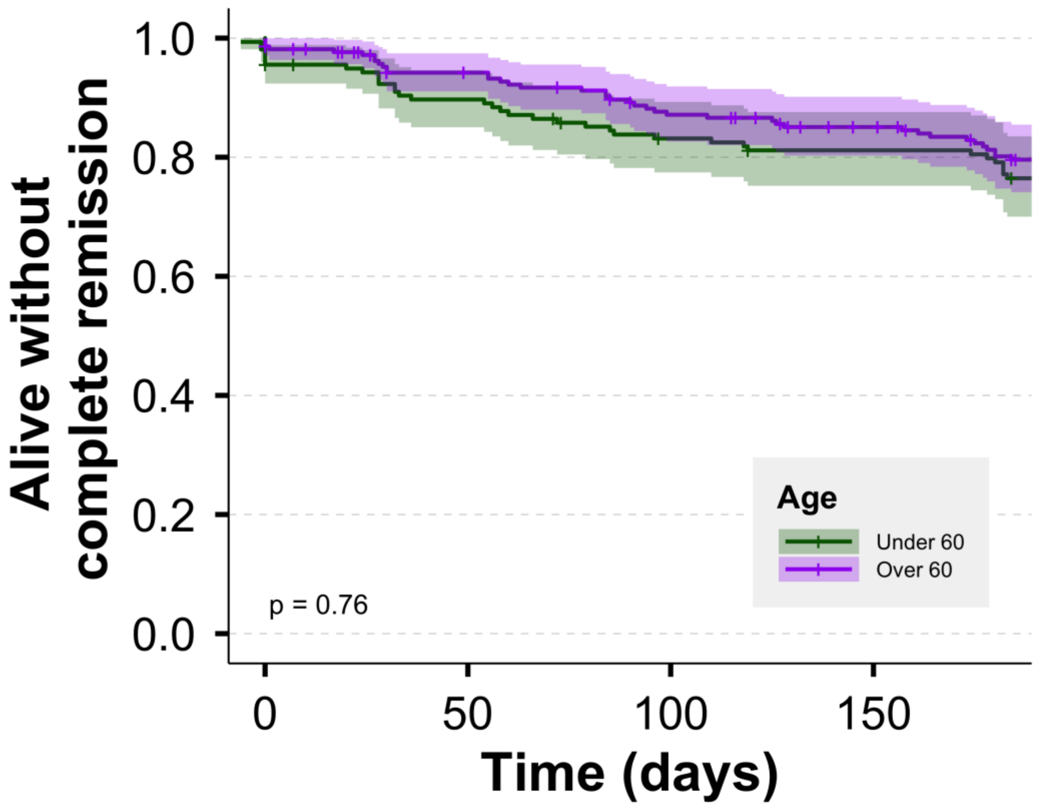

Number at risk

Under 60	154	142	135	127	121	121	119
Over 60	215	193	185	177	168	159	148

B

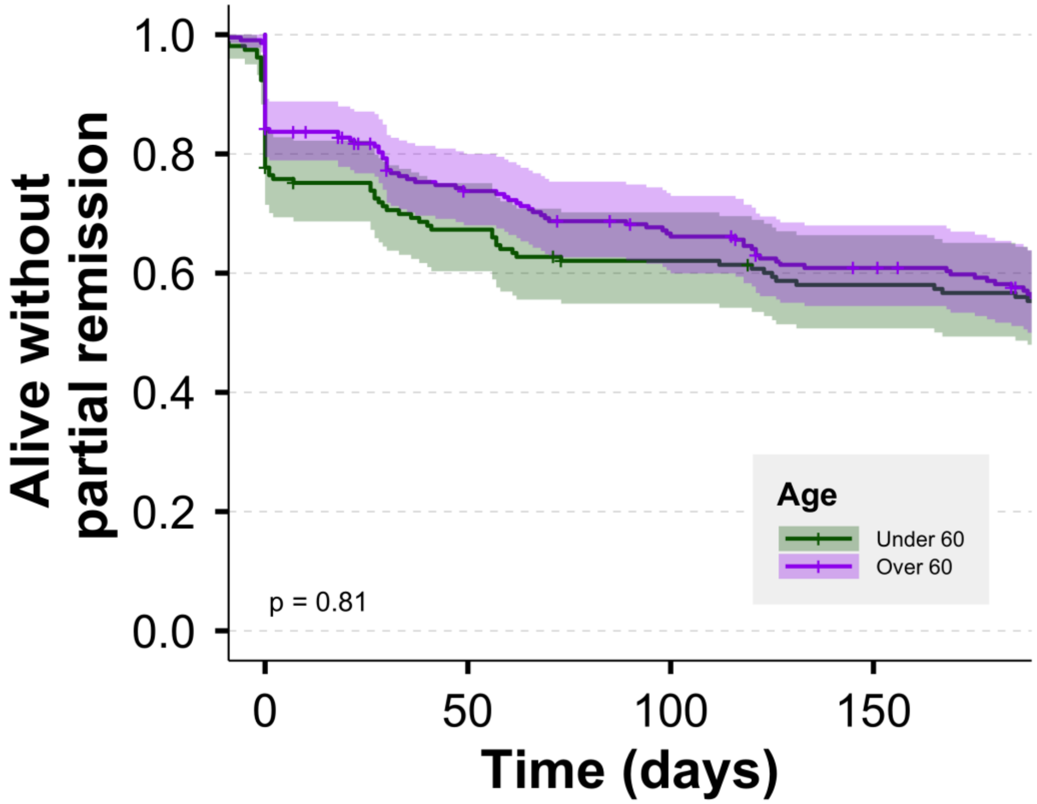

Number at risk

Under 60	145	109	98	93	91	86	84
Over 60	212	159	144	133	123	114	108

C

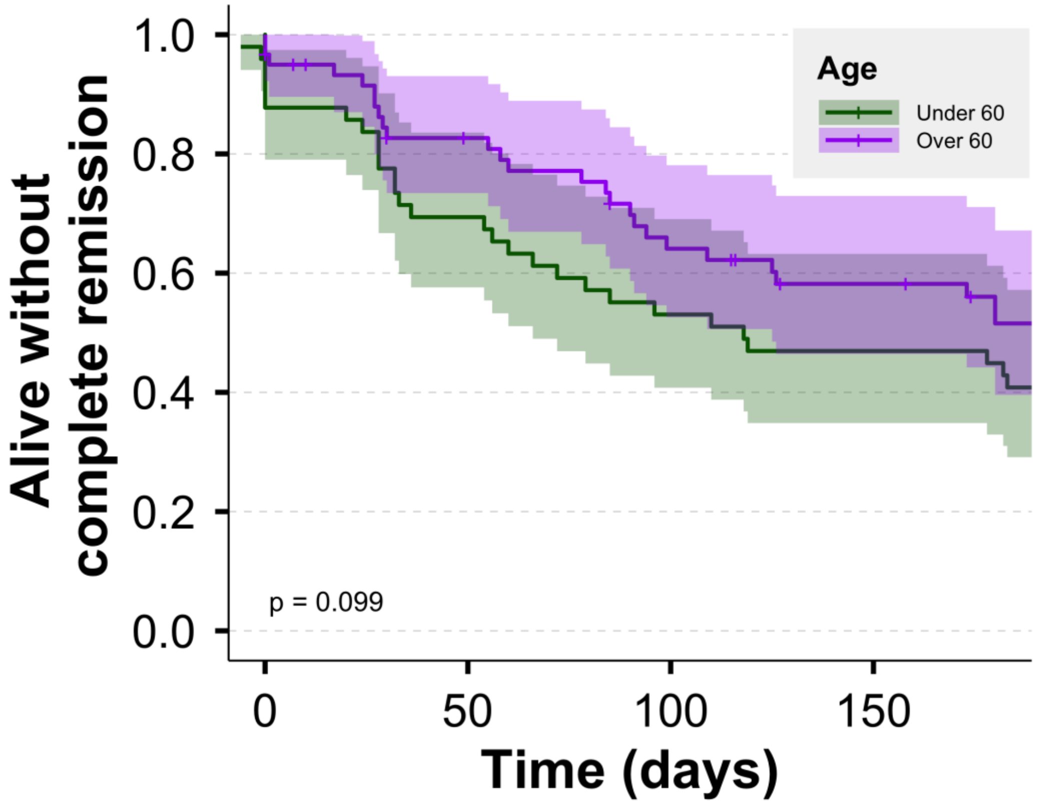

Number at risk

Under 60	47	38	32	27	23	23	22
Over 60	60	48	43	38	31	28	25

D

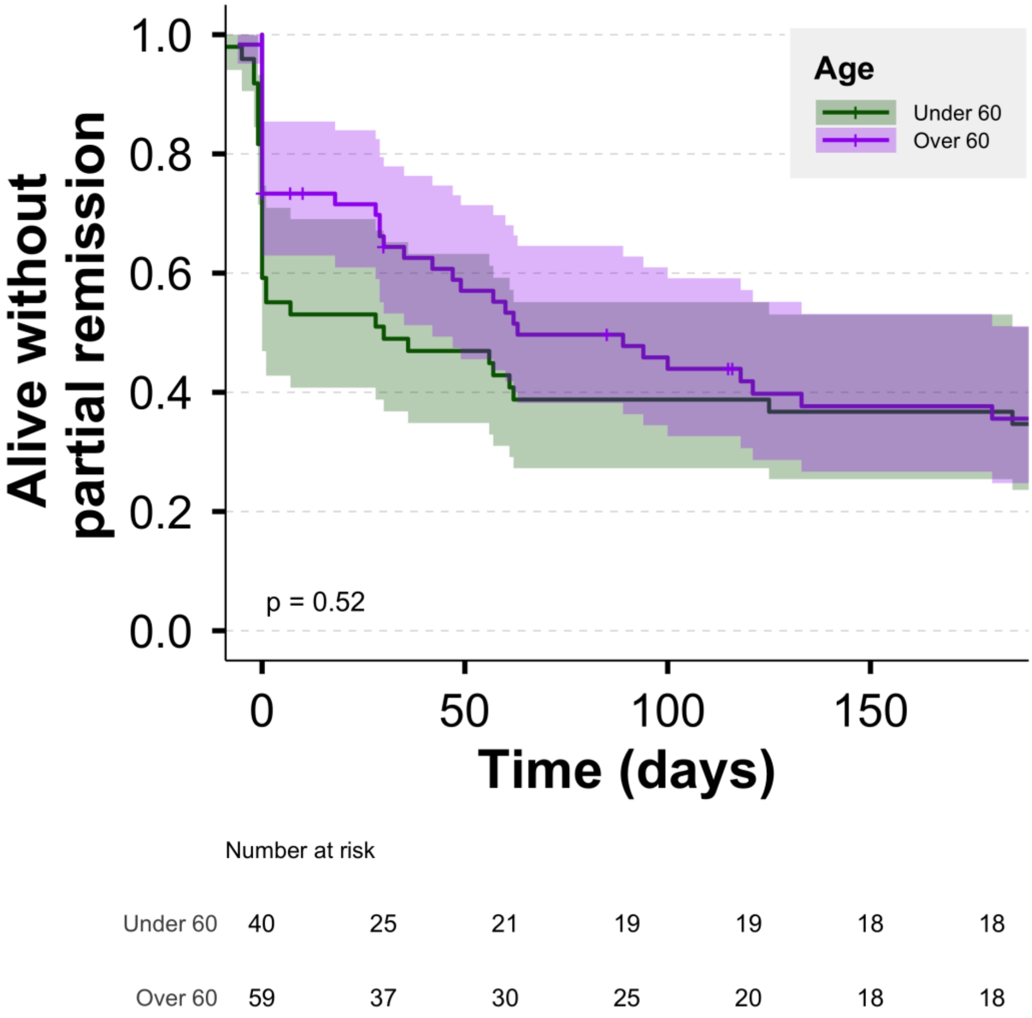
Alive without
partial remission
1.0
0.8
0.6
0.4
0.2
0.0
Age
Under 60
Over 60
p = 0.52
0
50
100
150
Time (days)
Number at risk
Under 60 40 25 21 19 19 18 18
Over 60 59 37 30 25 20 18 18

Figure S6

A

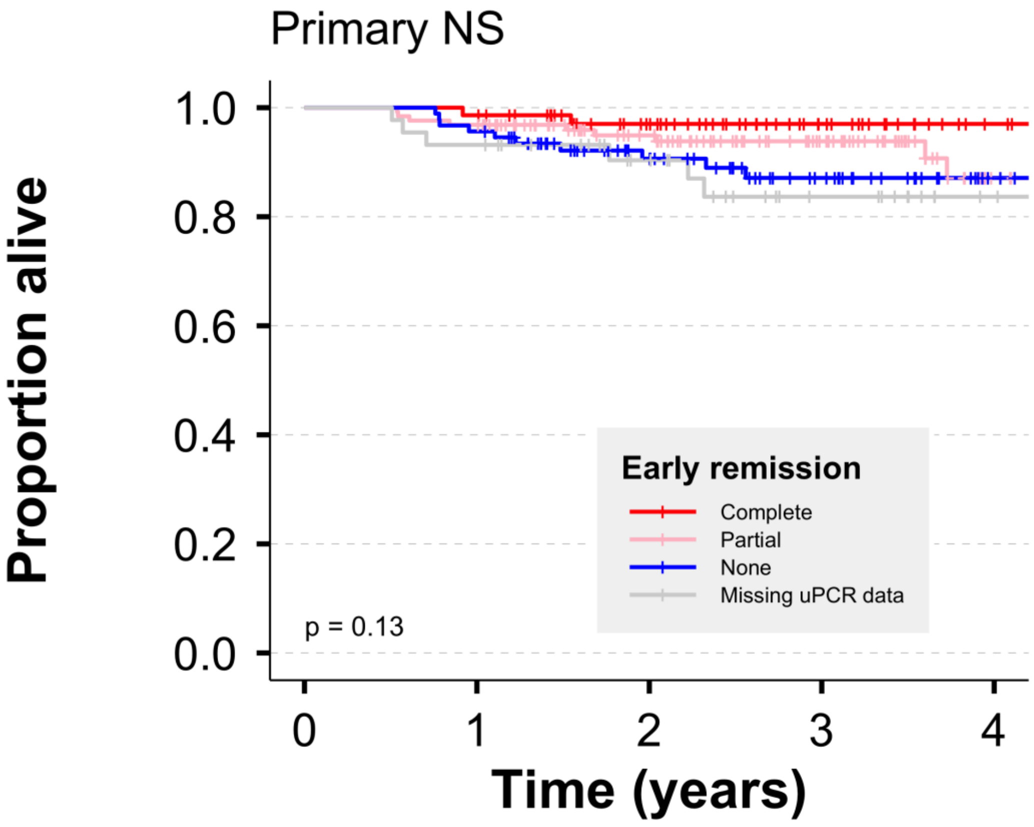

Number at risk

	0	1	2	3	4
Complete	72	71	53	31	15
Partial	127	123	90	55	17
None	92	88	61	38	13
Missing uPCR data	44	41	30	18	9

B

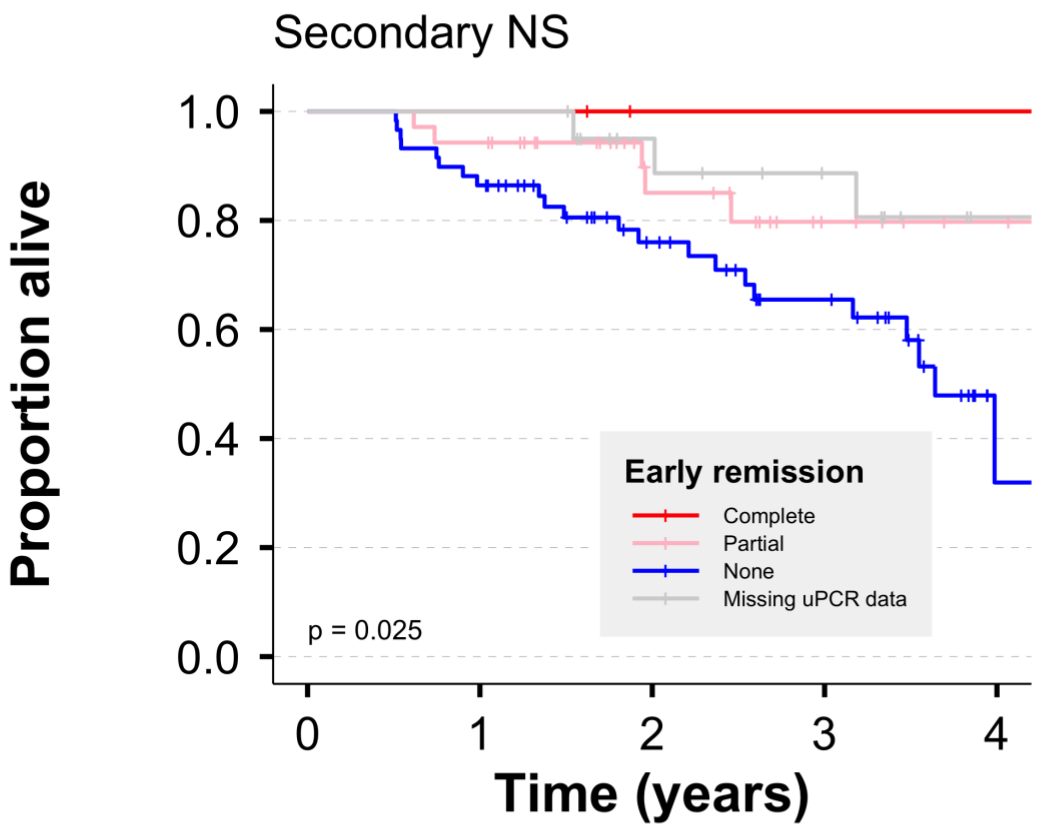

Number at risk

Complete	4	4	2	2	2
Partial	35	33	18	9	5
None	59	51	32	21	2
Missing uPCR data	21	21	15	11	4

C

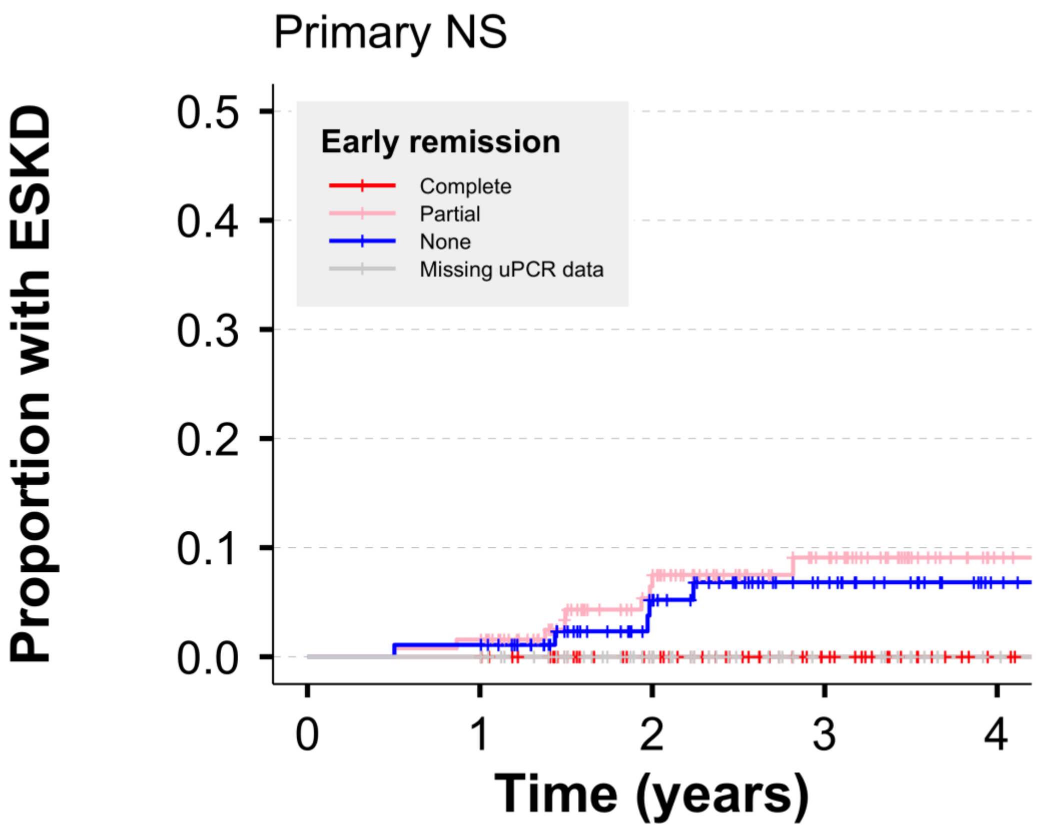

Number at risk

Complete	72	72	55	31	15
Partial	127	125	88	50	15
None	92	91	66	36	12
Missing uPCR data	44	44	33	18	9

D

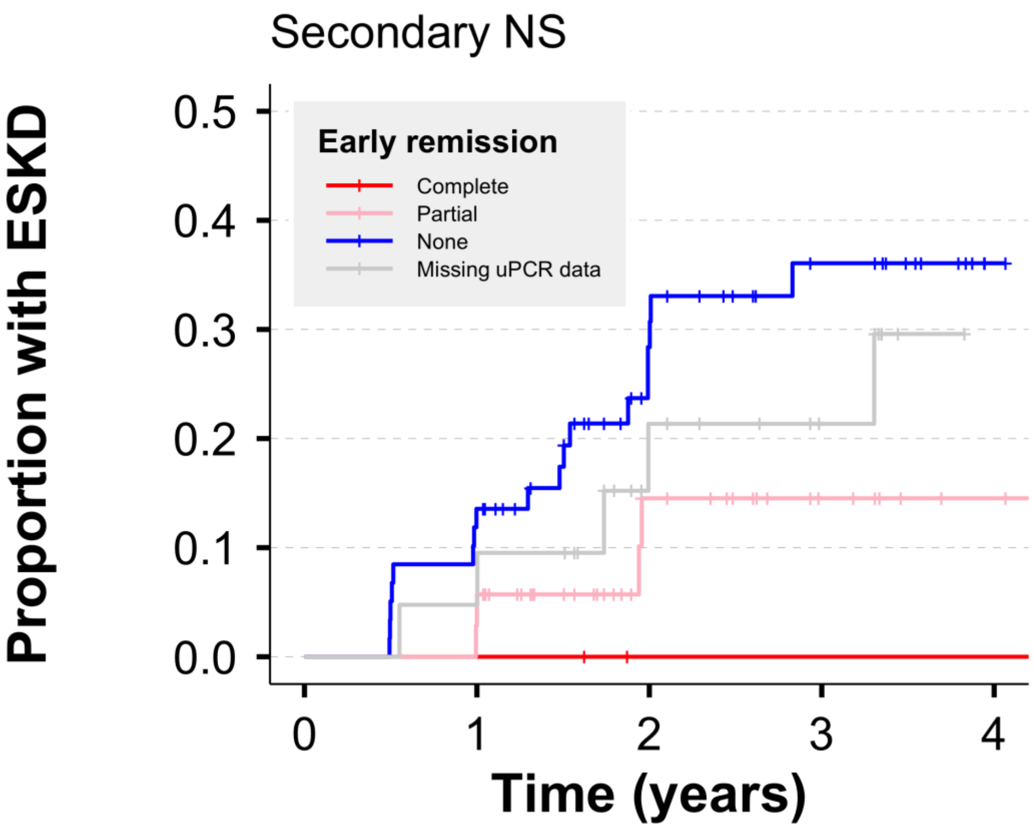

Number at risk

	0	1	2	3	4
Complete	4	4	2	2	2
Partial	35	34	18	9	6
None	59	51	31	20	3
Missing uPCR data	21	20	13	10	3

Figure S7

A

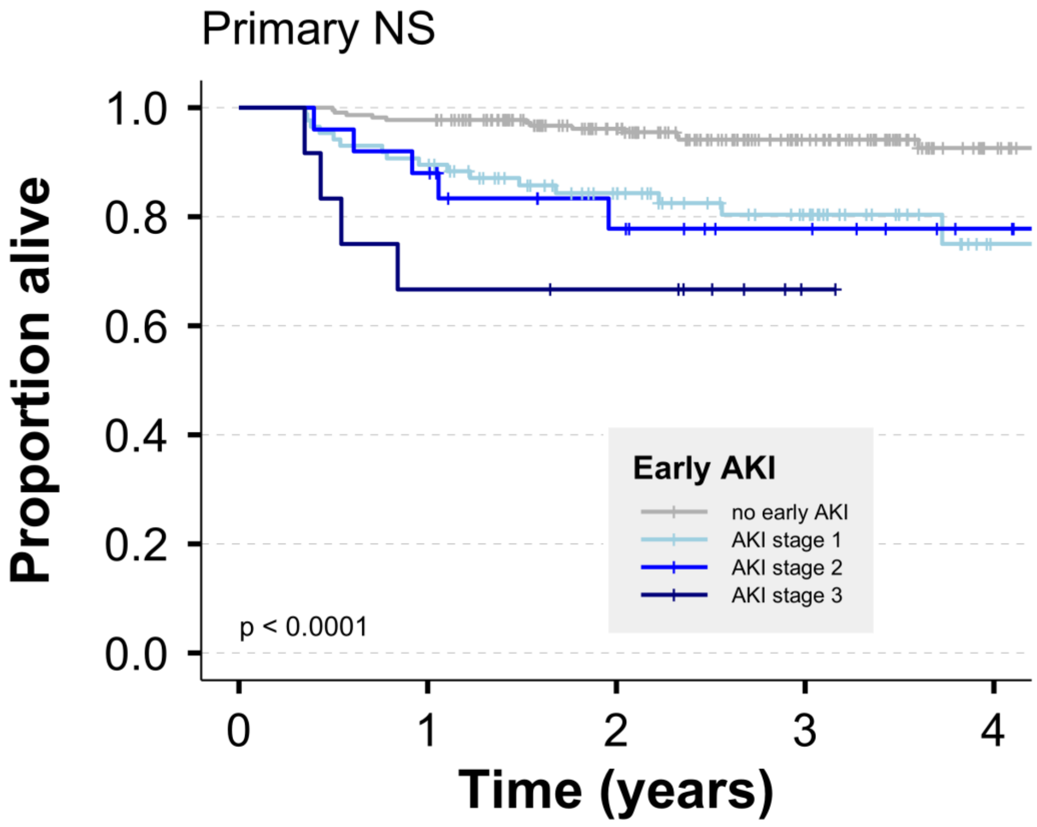

Number at risk

no early AKI	222	217	160	99	42
AKI stage 1	86	77	53	33	8
AKI stage 2	25	22	14	9	4
AKI stage 3	12	8	7	1	0

B

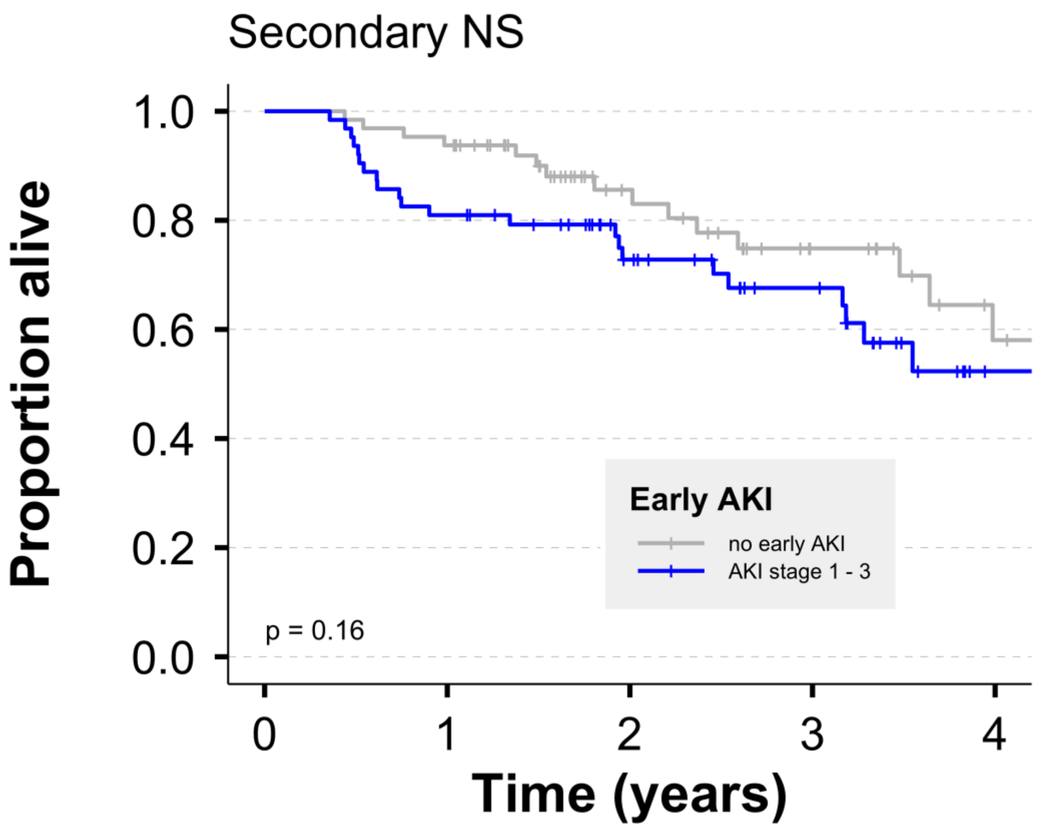

Number at risk

	0	1	2	3	4
no early AKI	64	60	33	19	9
AKI stage 1 - 3	63	51	33	22	4

C

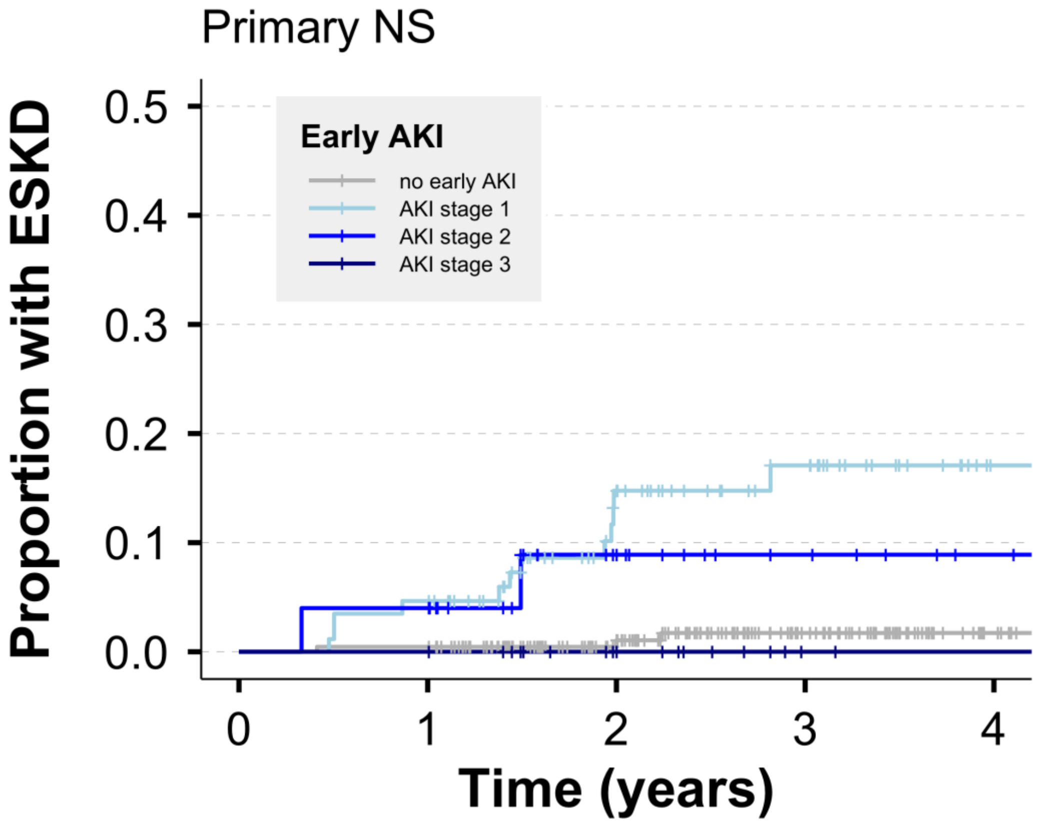

Number at risk

no early AKI	222	221	166	97	41
AKI stage 1	86	82	55	29	10
AKI stage 2	25	24	16	8	3
AKI stage 3	12	12	10	1	1

D

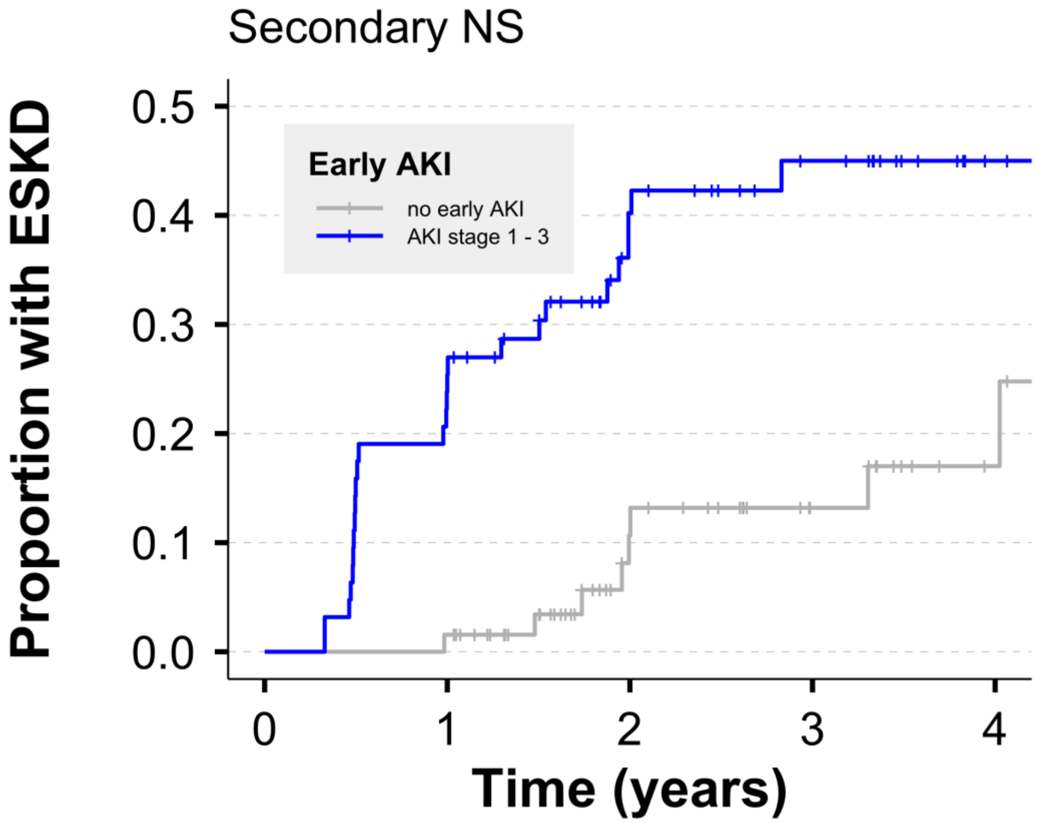

Number at risk

no early AKI	64	63	35	23	11
AKI stage 1 - 3	63	48	29	13	4

Supplemental References

1. The Scottish Renal Registry [Internet]. Available from: https://www.srr.scot.nhs.uk/Biopsy-Registry/Main.html [cited 2020 Jun 25]

2. McQuarrie EP, Mackinnon B, Young B, Yeoman L, Stewart G, Fleming S, Robertson S, Simpson K, Fox J, Geddes CC, Scottish Renal Biopsy Registry: Centre variation in incidence, indication and diagnosis of adult native renal biopsy in Scotland. *Nephrol Dial Transplant Off Publ Eur Dial Transpl Assoc - Eur Ren Assoc* 24: 1524–1528, 2009

3. Scottish Index of Multiple Deprivation 2020 - gov.scot [Internet]. Available from: https://www.gov.scot/collections/scottish-index-of-multiple-deprivation-2020/ [cited 2020 May 20]

4. Levey AS, Stevens LA, Schmid CH, Zhang YL, Castro AF, Feldman HI, Kusek JW, Eggers P, Van Lente F, Greene T, Coresh J, CKD-EPI (Chronic Kidney Disease Epidemiology Collaboration): A new equation to estimate glomerular filtration rate. *Ann Intern Med* 150: 604–612, 2009

5. Team NR of SW: National Records of Scotland [Internet]. Natl. Rec. Scotl. 2013 Available from: /statistics-and-data/statistics/statistics-by-theme/vital-events/general-publications/vital-events-reference-tables/2018/section-6-death-causes [cited 2020 May 21]

6. Thomas ME, Blaine C, Dawnay A, Devonald MAJ, Ftouh S, Laing C, Latchem S, Lewington A, Milford DV, Ostermann M: The definition of acute kidney injury and its use in practice. *Kidney Int* 87: 62–73, 2015

7. Cattran DC, Feehally J, Cook HT, Liu ZH, Fervenza FC, Mezzano SA, Floege J, Nachman PH, Gipson DS, Praga M, Glassock RJ, Radhakrishnan J, Hodson EM, Rovin BH, Jha V, Troyanov S, Li PKT, Wetzels JFM: Kidney disease: Improving global outcomes (KDIGO) glomerulonephritis work group. KDIGO clinical practice guideline for glomerulonephritis. *Kidney Int Suppl* 2: 139–274, 2012

8. R Core Team: R: A Language and Environment for Statistical Computing [Internet]. Vienna, Austria, R Foundation for Statistical Computing Available from: https://www.R-project.org/

9. Wickham H, Averick M, Bryan J, Chang W, McGowan LD, François R, Grolemund G, Hayes A, Henry L, Hester J, Kuhn M, Pedersen TL, Miller E, Bache SM, Müller K, Ooms J, Robinson D, Seidel DP, Spinu V, Takahashi K, Vaughan D, Wilke C, Woo K, Yutani H: Welcome to the tidyverse. *J Open Source Softw* 4: 1686, 2019

10. Kassambara A, Kosinski M, Biecek P: survminer: Drawing Survival Curves using "ggplot2" [Internet]. Available from: https://CRAN.R-project.org/package=survminer

11. Therneau TM: A Package for Survival Analysis in R [Internet]. Available from: https://CRAN.R-project.org/package=survival

12. Therneau T, Crowson C, Atkinson E: Multi-state models and competing risks. 29

13. Fine JP, Gray RJ: A Proportional Hazards Model for the Subdistribution of a Competing Risk. *J Am Stat Assoc* 94: 496–509, 1999

14. Gray B: cmprsk: Subdistribution Analysis of Competing Risks [Internet]. Available from: https://CRAN.R-project.org/package=cmprsk [cited 2020 Jun 25]

Modified STROBE Statement—checklist of items that should be included in reports of observational studies (Cohort/Cross-sectional and case-control studies)

	Item No	Recommendation	Author response
**Title and abstract**	1	(*a*) Indicate the study's design with a commonly used term in the title or the abstract	Yes ("registry")
		(*b*) Provide in the abstract an informative and balanced summary of what was done and what was found	Yes
**Introduction**			
Background/rationale	2	Explain the scientific background and rationale for the investigation being reported	Yes
Objectives	3	State specific objectives, including any prespecified hypotheses	Yes – including our pre-specified analysis protocol
**Methods**			
Study design	4	Present key elements of study design early in the paper	Yes
Setting	5	Describe the setting, locations, and relevant dates, including periods of recruitment, exposure, follow-up, and data collection	Yes
Participants	6	(*a*) *Cohort study*—Give the eligibility criteria, and the sources and methods of selection of participants. Describe methods of follow-up<br>*Case-control study*—Give the eligibility criteria, and the sources and methods of case ascertainment and control selection. Give the rationale for the choice of cases and controls<br>*Cross-sectional study*—Give the eligibility criteria, and the sources and methods of selection of participants	Yes (cohort study)
Variables	7	Clearly define all outcomes, exposures, predictors, potential confounders, and effect modifiers. Give diagnostic criteria, if applicable	Yes
Data sources/ measurement	8*	For each variable of interest, give sources of data and details of methods of assessment (measurement).	Yes
Bias	9	Describe any efforts to address potential sources of bias	Yes

Study size	10	Explain how the study size was arrived at (if applicable)	Yes (we included all patients over a set time period)
Quantitative variables	11	Explain how quantitative variables were handled in the analyses. If applicable, describe which groupings were chosen and why	Yes
Statistical methods	12	(*a*) Describe all statistical methods, including those used to control for confounding	Yes
		(*b*) Describe any methods used to examine subgroups and interactions	Yes
		(*c*) Explain how missing data were addressed	Yes
		(*d*) *Cohort study*—If applicable, explain how loss to follow-up was addressed<br>*Case-control study*—If applicable, explain how matching of cases and controls was addressed<br>*Cross-sectional study*—If applicable, describe analytical methods taking account of sampling strategy	Yes
		(*e*) Describe any sensitivity analyses	Yes
**Results**			
Participants	13*	(a) Report numbers of individuals at each stage of study—eg numbers potentially eligible, examined for eligibility, confirmed eligible, included in the study, completing follow-up, and analyzed	Yes
		(c) **Use of a flow diagram**	No – but happy to supply one
Descriptive data	14*	(a) Give characteristics of study participants (eg demographic, clinical, social) and information on exposures and potential confounders	Yes
		(b) Indicate number of participants with missing data for each variable of interest	Yes
		(c) *Cohort study*—Summarise follow-up time (eg, average and total amount)	Yes
Outcome data	15*	*Cohort study*—Report numbers of outcome events or summary measures over time	Yes

		Case-control study—Report numbers in each exposure category, or summary measures of exposure	
		Cross-sectional study—Report numbers of outcome events or summary measures	
Main results	16	(*a*) Give unadjusted estimates and, if applicable, confounder-adjusted estimates and their precision (eg, 95% confidence interval). Make clear which confounders were adjusted for and why they were included	Yes
Other analyses	17	Report other analyses done—eg analyses of subgroups and interactions, and sensitivity analyses	Yes
**Discussion**			
Key results	18	Summarise key results with reference to study objectives	Yes
Limitations	19	Discuss limitations of the study, taking into account sources of potential bias or imprecision. Discuss both direction and magnitude of any potential bias	Yes
Interpretation	20	Give a cautious overall interpretation of results considering objectives, limitations, multiplicity of analyses, results from similar studies, and other relevant evidence	Yes
Generalisability	21	Discuss the generalisability (external validity) of the study results	Yes

*Give information separately for cases and controls in case-control studies and, if applicable, for exposed and unexposed groups in cohort and cross-sectional studies.

Note: An Explanation and Elaboration article discusses each checklist item and gives methodological background and published examples of transparent reporting. The STROBE checklist is best used in conjunction with this article (freely available on the Web sites of PLoS Medicine at http://www.plosmedicine.org/, Annals of Internal Medicine at http://www.annals.org/, and Epidemiology at http://www.epidem.com/). Information on the STROBE Initiative is available at www.strobe-statement.org.
